# Understanding the Spatial Scale of Genetic Connectivity at Sea: Unique Insights from a Land Fish and a Meta-Analysis

**DOI:** 10.1371/journal.pone.0150991

**Published:** 2016-05-19

**Authors:** Georgina M. Cooke, Timothy E. Schlub, William B. Sherwin, Terry J. Ord

**Affiliations:** 1 Evolution and Ecology Research Centre, School of Biological, Earth and Environmental Sciences, University of New South Wales, Kensington 2052 NSW, Australia; 2 The Australian Museum, Australian Museum Research Institute, Ichthyology, 6 College Street, Sydney NSW 2010, Australia; 3 Sydney School of Public Health, Sydney Medical School, University of Sydney, 2006 NSW, Australia; Chinese Academy of Sciences, CHINA

## Abstract

Quantifying the spatial scale of population connectivity is important for understanding the evolutionary potential of ecologically divergent populations and for designing conservation strategies to preserve those populations. For marine organisms like fish, the spatial scale of connectivity is generally set by a pelagic larval phase. This has complicated past estimates of connectivity because detailed information on larval movements are difficult to obtain. Genetic approaches provide a tractable alternative and have the added benefit of estimating directly the reproductive isolation of populations. In this study, we leveraged empirical estimates of genetic differentiation among populations with simulations and a meta-analysis to provide a general estimate of the spatial scale of genetic connectivity in marine environments. We used neutral genetic markers to first quantify the genetic differentiation of ecologically-isolated adult populations of a land dwelling fish, the Pacific leaping blenny (*Alticus arnoldorum*), where marine larval dispersal is the only probable means of connectivity among populations. We then compared these estimates to simulations of a range of marine dispersal scenarios and to collated *F*_ST_ and distance data from the literature for marine fish across diverse spatial scales. We found genetic connectivity at sea was extensive among marine populations and in the case of *A*. *arnoldorum*, apparently little affected by the presence of ecological barriers. We estimated that ~5000 km (with broad confidence intervals ranging from 810–11,692 km) was the spatial scale at which evolutionarily meaningful barriers to gene flow start to occur at sea, although substantially shorter distances are also possible for some taxa. In general, however, such a large estimate of connectivity has important implications for the evolutionary and conservation potential of many marine fish communities.

## Introduction

Genetic exchange among individuals and between populations—i.e. genetic connectivity—is important for the evolutionary dynamics of species across all spatial and temporal scales, from a local to regional level and from thousands to millions of years. Indeed, there has been enormous interest in estimating gene flow across space and time and this information has been used to understand biological and evolutionary processes like adaptation, biogeographic history and speciation [1]. In addition, estimations of gene flow are being used to improve the design and implementation of management strategies that maximise genetic fitness among threatened populations through the appropriate spatial placement of reserves or wildlife corridors [2]. However, for organisms in which dispersal is characterized by small gametes or offspring—e.g. marine fish with pelagic larvae—accurate predictions of the degree to which populations are impacted by dispersal and subsequent connectivity (also known as ‘demographic connectivity’) have been difficult to make. This is partly because the nature of marine ecosystems often precludes the direct measure of the number and type of individuals moving or interacting among populations (also known as demographic connectivity e.g. through tagging and mark-re-capture [3, 4]). Indeed, for many fish species, the spatial scale of connectivity is set by pelagic larvae that may be dispersed by highly advective ocean currents for several days to weeks before settlement, which might then be followed by either a sedentary or migratory adult phase [5–9]. This means that populations of marine fish appear to have high connectivity across very large spatial scales often upwards of 300 km [9–12]. Yet, despite the apparent capacity for high genetic exchange at sea, the behaviour of larval fish can also limit dispersal. In particular, larvae are capable of highly directional swimming that can minimize the influence of mean ambient currents [13] and can result in self-recruitment to natal habitats despite oceanic currents. This in turn reduces connectivity to much smaller spatial scales [13–16]. Consequently, predicting the magnitude and geographic scale of connectivity of fish in the marine environment has been a notoriously difficult task.

Given this difficulty in measuring marine connectivity, indirect methods such as the genetic estimation of population structure and gene flow have been employed [9]. Because differentiation of neutral genes among populations is dependent on gene flow, differentiation is expected to be affected by dispersal ability, restriction of population size and the extent of isolation and habitat connectivity. Therefore, genetic analysis of population structure using Wright’s *F*_ST_ ([17]; and its analogues) has been a common genetic method for estimating the spatial scale and magnitude of connectivity in the marine environment [9, 18]; e.g. the relationship between pelagic larval dispersal in distance (PLD) and *F*_ST_ [19, 20, 21]. One such model is Wright’s [17] island model in which there is equal dispersal between all pairs of local populations (such equal dispersal is unlikely in most systems, but the model nevertheless provides useful predictions that can be used to benchmark data). An alternative model, known as isolation by distance (IBD; [22]), has higher dispersal between closer localities, such that closer populations will be more similar at neutral genetic markers. In other words, isolation by distance predicts that pairwise genetic divergence (*F*_ST_ or alternatives) among populations will be positively correlated with geographic distance (e.g. [23]). As a result of the arguably more realistic “stepping stone” scenario of IBD theory, it has been a frequently utilized model in studies of marine connectivity [12, 24, 25]. Despite this, there is considerable debate surrounding the relationship between dispersal and *F*_ST_ [26, 27], as well as the effectiveness of *F*_ST_ as a measure of genetic structure compared to its analogues (e.g. [28–31]). While many of these authors have attempted to compensate for these problems providing alternative methods to measure genetic structure, the ubiquity of the *F*_ST_ method in the marine connectivity literature [20], and the fact that it arguably accounts for mutation processes better than its analogues [31], means that it continues to be one of the most valuable metrics for the quantification of genetic connectivity in marine fish and subsequent comparison among published data.

**Fig 1:**
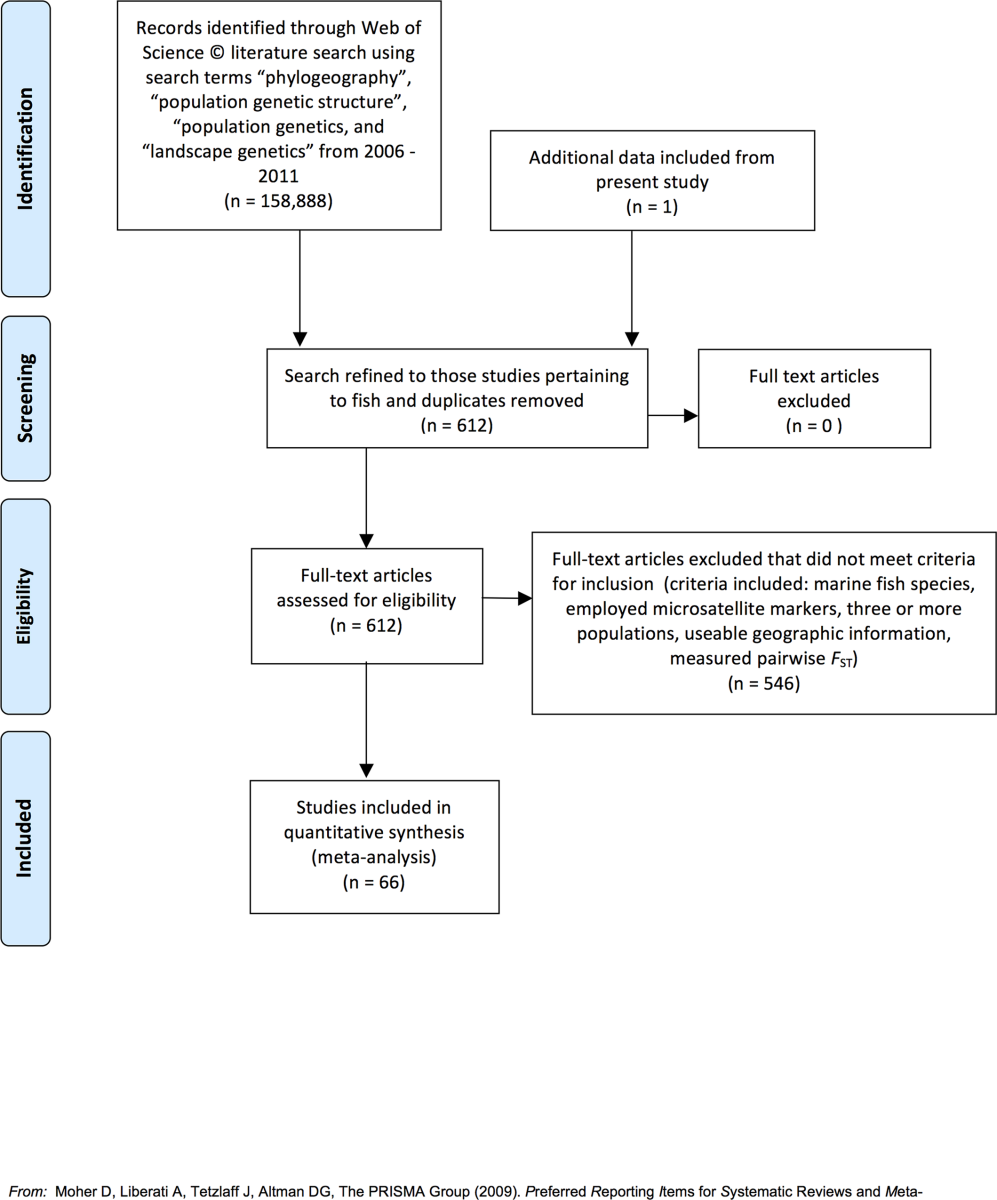
PRISMA 2009 Flow diagram. Depicts the selection process of studies included in the meta-analysis.

**Fig 2:**
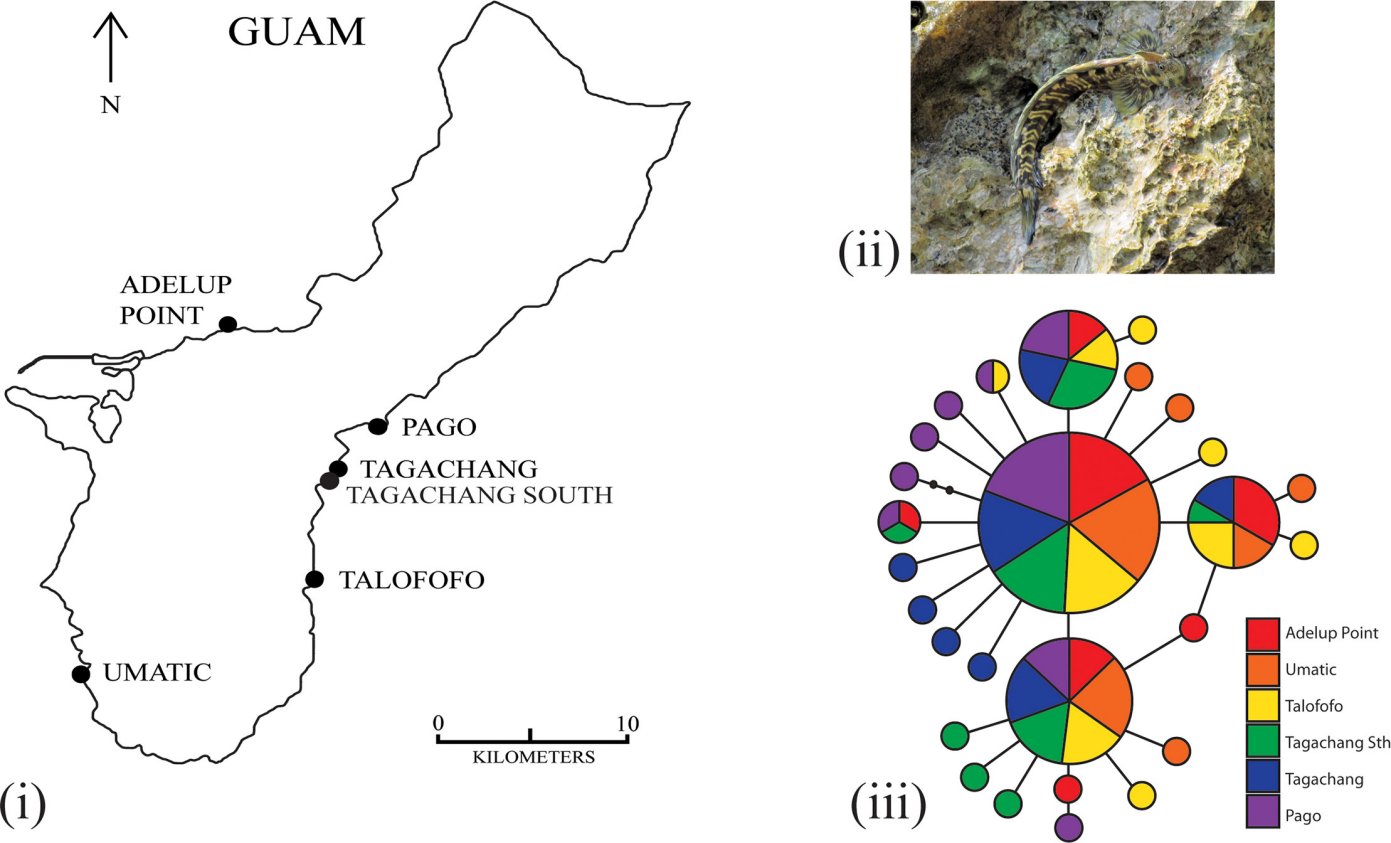
Sampling localities and haplotype network. The (i) sampling localities of *Alticus arnoldorum* around the island of Guam, site abbreviations as in Table 1, (ii) *A*.*arnoldorum* (photo G Cooke), and (iii) results from the haplotype network based on 120 mtDNA ATPase 6 and 8 sequences. Each circle denotes a unique haplotype, the area of the circle is proportional to its frequency in the sample, and the shade of the circle represents its sampling location.

In this study we combined several complementary approaches combined with a meta-analysis (Fig 1; S1 Fig) to better understand and predict the spatial scale of genetic connectivity in marine fishes. First, we examined connectivity in the context of population demography and fine scale genetic structure among populations of an unusual fish, the Pacific leaping blenny (*Alticus arnoldorum*) found on the Micronesian island of Guam (Fig 2i and 2ii).

*Alticus arnoldorum* live their adult life out of water at high densities along the supralittoral zone [32]. They have enhanced cutaneous respiration [33–35] and terrestrial locomotor abilities that allow them to move about with extreme agility on land [36]. Adult fish are highly territorial and are rarely seen to voluntarily return to water [32]. The fish are susceptible to desiccation at low tides and displacement from perches by violent wave action at high tide. This results in a brief temporal window at mid-tide during which most activity is restricted (e.g. foraging) and more generally confines these land fish to the supralittoral splash zone on the island [32]. Given that suitable habitat for this fish around the coast of Guam is discontinuous–the rocky outcrops on which they live are interspersed by large beaches that represent a formidable barrier to these fish–adult dispersal among populations is virtually impossible. However, the larvae of *A*. *arnoldorum* are almost certainly pelagic (settlement occurs around 28 days, Platt and Ord, unpublished), and are the most likely means by which individuals might be exchanged among populations. Because of this, *A*. *arnoldorum* provides a good opportunity to quantify the geographic extent of connectivity among populations that results primarily from the movement of marine larvae. This can be extremely difficult to achieve in genetic studies of population structure in the marine environment that sum the results of larval and adult dispersal (for rare examples see [37–40]).

We compared our estimates of population genetic differentiation of *A*. *arnoldorum* on Guam to genetic differentiation data from simulations that assumed a range of realistic marine dispersal scenarios for this species. The simulation used a spatial matrix of the inter-tidal zone around Guam and used various density-dependent models of dispersal. Thus, we were able to evaluate the extent to which a primarily larval-dispersed marine fish exhibited predictable or unexpected levels of population genetic differentiation.

Second, we placed these findings from the larval-dispersing *A*. *arnoldorum* into its broadest context by obtaining a general estimate of connectivity among marine fishes (that might reflect dispersal via larvae, adults or both) using a meta-analysis of *F*_ST_ and distance data from published literature. Estimates of gene flow using *F*_ST_ have been documented by hundreds of studies for an equally vast number of spatial scales and different organisms including fishes. We took advantage of this enormous resource to estimate the rate at which gene flow is curbed by distance in marine fish across all environments and spatial scales. In doing so, we generalised the extent to which *F*_ST_ increases with distance and the magnitude of connectivity in marine fish globally.

**Fig 3:**
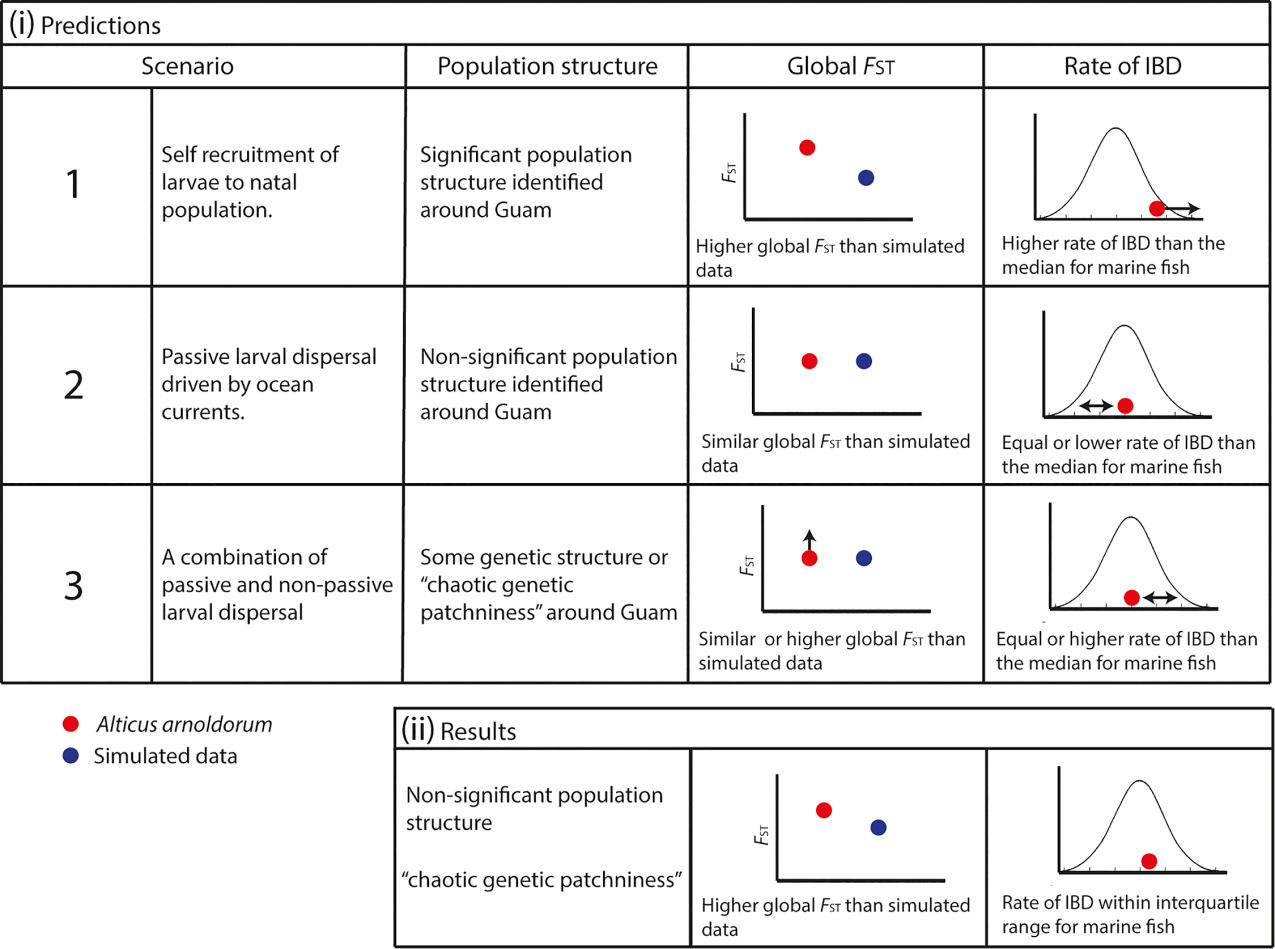
**Scenarios of how the behaviour of larvae might impact genetic connectivity** (i) Predictions based on realistic dispersal scenarios of *Alticus arnoldorum* incorporating empirical, simulated and meta-analyses results. (ii) Schematic illustrating the results from *Alticus arnoldorum* compared to the simulated and meta-analysis results.

By integrating an empirical study, simulations and a meta-analysis (Fig 1), our overarching goal was to estimate the spatial scale of genetic connectivity at sea for fish and evaluate the extent to which pelagic larval dispersal in fish impacts genetic connectivity among populations that are otherwise isolated from one another by ecological barriers to adult dispersal. To this end, we tested three possible scenarios of how the behaviour of larvae might impact genetic connectivity (Fig 3):

1. If self-recruitment of larvae to natal habitats is high in *A*. *arnoldorum*, with little to no adult dispersal, populations should exhibit higher global *F*_ST_ than the simulated data (that models dispersal among populations under a general IBD model) and low connectivity between populations that are geographically close (i.e. <100km, or significant *F*_ST_ between populations around Guam). This outcome would indicate a more terrestrial mode of dispersal. Indeed, terrestrial animals such as mammals that cannot disperse in their earliest developmental stage generally have higher global *F*_ST_ than larval dispersers like fish [41]. In some cases, significant genetic structure can be observed in small mammals across distances as little as <10km (e.g. [42]). Given this, the rate of IBD in *A*. *arnoldorum* should be much higher than the median rate for marine fish collated in our meta-analysis if combined larval and adult dispersal results in high genetic connectivity in the marine environment (i.e. [9–12]).
2. Alternatively, connectivity among populations of *A*. *arnoldorum* might result from passive larval dispersal driven by ocean eddies and currents around Guam, followed by a sedentary adult phase (i.e., a transition from a marine to land environment where adult populations are subsequently ecologically isolated from one another). In this scenario, a Lagrangian larval dispersal model [43] that assumes a one month pelagic larval period similar to that of *A*. *arnoldorum* predicts dispersal distances of up to 300km (~10km/day). As the circumference of Guam falls within this distance (~150 km), there should be no significant population structure among populations of *A*. *arnoldorum*. Instead, the global *F*_ST_ of *A*. *arnoldorum* should be similar to simulations that assume high dispersal scenarios (greater than the maximum distance between any two populations, i.e. 300km) with a rate of IBD in *A*. *arnoldorum* being equal to, or less than, the median rate for marine fish estimated by our meta-analysis.
3. Finally, connectivity among populations of *A*. *arnoldorum* might be a combination of passive and active larval dispersal, followed by a sedentary adult phase (see scenario 2 above). Such a pattern could occur if natural selection is acting on a local level either before or after settlement due to ecological differences between sites (e.g., see [44]). In this situation we expect to see some genetic structure or ‘chaotic genetic patchiness’ in which there is small-scale, unpatterned genetic heterogeneity among local populations [45, 46], which may not necessarily be correlated with distance. Here, some cohesion or active dispersal of larvae between sites may skew the relationship between geographic distance and genetic divergence. Furthermore, global *F*_ST_ should be similar to or higher than simulations that assume moderate dispersal scenarios (greater than or equal to the maximum distance between any two populations), and the rate of IBD in *A*. *arnoldorum* should be equal to, or greater than, the median rate of *F*_ST_ and distance for marine fish from our meta-analysis.

## Materials and Methods

### Sampling and genetic methods

This study was carried out following procedures set by the University of New South Wales Animal Care and Ethics Committee in protocol #11/36b, initially approved on the 10th March 2011 and most recently reviewed on the 28th February 2013. Specimens were euthanized by first anaesthetizing fish using clove oil and then storing them under ice. No permits or approvals were required to collect specimens on Guam, and no work was conducted on private or protected land. All data from this publication have been archived in the Dryad Digital Repository (doi:10.5061/dryad.v63g0) and Genbank (KU922092-KU922117).

Thirty-four individual *Alticus arnoldorum* fish (17 male and 17 female) were collected each from six field locations around Guam (total sample size of 204 adult fish; Table 1). Sampling locations ranged from just ~200 m apart (coastal distance), being separated by a single beach (Taga’chang north and Taga’chang south; Fig 2), to ~90 km apart (Pago to Adelup Point; Fig 2) where sites were separated by numerous inhospitable terrestrial barriers (e.g., beaches, dry rocks and shrubland). Fish were caught using hand nets, euthanized, and muscle tissue was dissected and preserved in 20% DMSO in a saturated NaCl_2_ solution. DNA was extracted using a DNeasy blood and tissue extraction kit (Qiagen) and data were obtained from both the mitochondrial (mtDNA) and nuclear genomes. The mtDNA adenosine triphosphate subunits 6 and 8 (ATPase 6, 8) were amplified via polymerase chain reaction (PCR) for 20 samples per site using primers ATP8.2 and CO3.2 [47] with PCR conditions as in Cooke et al. [48]. PCR products were cleaned using Exosapit (Affymetrix), and sequenced by Macrogen on a 3730XL DNA sequencer. For the nuclear data set (number *(n)* = 204) we developed 17 novel microsatellite loci for *A*. *arnoldorum* using 454 next generation sequencing technology following Gardner et al [49]. A minimum of 500ng of DNA was sequenced in 1/8^th^ of a PicoTiter plate at the Australian Genome Research Facility (AGRF, www.agrf.com.au) on a Roche GL FLX (454) system. QDD was then used to detect microsatellites in the 454 output and to design primers. 1724 sequences containing putative microsatellite motifs with a minimum number of five repeats were identified. Of these, we selected 20 of the best loci for PCR trials, resulting in 17 polymorphic loci (primers: S1 Table). PCR amplification was performed in 10μL reactions {1 × buffer (Promega), 2 mM MgCl_2_, 0.05 mM of each dNTP, 10 μm of each primer and 0.5 U *Taq* polymerase (Promega)} with an initial denaturing at 95°C for 60 s, followed by a 65–53°C touch-down, ending with 30 cycles of 95°C for 15 s, 53°C for 15s and 72°C for 30 s with a final extension of 70°C for 5 min. Multiplexed PCR products using labelled primers (S1 Table) were run at the Australian Genome Research Facility on a 3730xl sequencer and the electropherograms were analysed and scored manually using GeneMapper version 4.1 (Applied Biosystems).

**Table 1:** Sampling localities, sample sizes and genetic diversity at mtDNA and microsatellite markers (PWD, pair wise differences).

Population	Label	Coordinates	Sample size (mtDNA/ μsats)	No. haplotypes	Mean no. PWD	Nucleotide diversity (%)
Adelup Point	AP	N 13°28.873', E 144°43.732'	20/34	7	1.178947	0.14
Umatac	UM	N 13°17.764', E 144°39.633'	20/34	7	1.110526	0.1319
Talofofo	TF	N 13°20.684', E 144°46.282'	20/34	9	1.5	0.1781
Taga’chang South	TS	N 13°24.220', E 144°46.907'	20/34	8	1.315789	0.1563
Taga’ chang	TC	N 13°24.403', E 144°46.969'	20/34	8	1.194737	0.1419
Pago	PG	N 13°25.664', E 144°47.943'	20/34	8	1.405263	0.1669

### Sequence analysis and demographic history

The 120 mitochondrial ATPase 6 and 8 sequences were aligned using Geneious v.5.6. (Biomatters, http://www.geneious.com) and genealogical relationships among individuals were investigated using the coalescent-based approach in TCS [50, 51]. Sequence diversity was estimated as haplotypic diversity and nucleotide diversity [52] per population in Arlequin 3.5.1.2 [53].

Demographic or selection history of the entire mitochondrial dataset was assessed by computing a mismatch distribution in Arlequin. Mismatch analysis tests for the agreement of the data with a model of demographic expansion [53, 54]. Fu’s [55] test of demographic history or selective neutrality was also employed to assess the signal of expansion in the data set. In the event of demographic expansion or directional selection, large negative *F*_S_ values are generally observed. We also assessed the demographic history of the *A*. *arnoldorum* on Guam with a Bayesian Skyline Plot (BSP; [56]) modelled in BEAST v1.7.2 [57] using the mitochondrial ATPase 6 and 8 sequence data. A BSP is the posterior distribution of the effective population size through time generated using a standard Markov Chain Monte Carlo (MCMC) sampling procedure assuming a single panmictic population. For the analysis, we specified a strict molecular clock with a fixed mutation rate of 1.4% per million years [47] and a GTR model of sequence evolution. These parameters were chosen because systematic rate heterogeneity is not expected in intraspecific data. The number of grouped individuals was set to five and two analyses were run for 100 million generations, sampling every 1000. We combined the independent runs and all effective sample sizes (ESS) were >200. Tracer v1.5 [58] was then used to analyse the runs and generate the skyline plots.

### Population genetic structure

For the mitochondrial data set, pairwise population genetic structure was calculated as Φ_ST_ [59] and the degree of population structure was explored with a hierarchical analysis of molecular variance (AMOVA) in Arlequin [53]. Isolation by distance (IBD; [60]) was investigated using a Mantel permutation test [60] of the association between genetic distance (Φ_ST_) and geographic distance, either direct (Euclidian) or coastal distance in Arlequin [53].

For the microsatellite dataset, the 17 microsatellite loci were tested for departures from Hardy-Weinberg equilibrium (HW) in Arlequin and linkage disequilibrium was assessed using Genepop [60, 61]. Microchecker [62] was then used to determine whether any observed departures from HW at each locality was due to null alleles, allele dropout or allele stuttering. The extent of inbreeding was also estimated using the IIM (individual inbreeding model) approach with 10,000 iterations implemented in INEst [63]. This method discriminates between heterozygote deficits due to null alleles, and deficits due to other causes such as inbreeding. It allows the calculation of unbiased estimates for a multilocus average inbreeding coefficient (*F*_IS_) in the presence of null alleles at proportions (*p*_n_). We estimated genetic diversity at each locality as number of alleles per locus, allelic richness, and Wright’s inbreeding coefficient (*F*_IS_), using the software FSTAT [64] and expected and observed heterozygosity using Arlequin [53].

Pairwise genetic differentiation (*F*_ST_) of microsatellites among populations was estimated and tested for significance with 10,000 permutations using Arlequin [53]. In addition, we calculated G’_ST_est_ [28] and D_est_ [29] using SMOGD v.1.2.5 [65] and their correlation with *F*_ST_ was tested using a linear regression [66]. We also calculated Shannon’s information index of population subdivision (^*S*^*H*_*UA*_) which is thought to provide another robust estimation of genetic exchange in addition to *F*_ST_ [27, 30]_,_ for pairwise population comparisons in Genalex [67].

Structure v2.3.4 was used to identify the presence of populations or genetic clusters in *A*. *arnoldorum* on Guam based on microsatellite data. The most likely value of *K*, the number of clusters, was determined by plotting the mean natural log (Ln) probability of the data versus *K* over multiple runs and change in K (∆K) following Evanno et al. [68] with 1,000,000 MCMC repetitions and a burn in of 10,000 iterations. In each case, prior population information was not used, and correlated allele frequencies and admixed populations were assumed. Mantel permutation tests [60] were also used with the microsatellite data to test for the association between genetic distance (*F*_ST_) and direct and coastal distance (IBD; [22]) in Arlequin [53]. Spatial autocorrelation analysis as calculated in Genalex [67] was then used to identify the scale of spatial genotypic structure among *A*. *arnoldorum* populations around Guam. The autocorrelation coefficients of multilocus microsatellite genotypes (*r*) was calculated for individuals sampled in the same locality (distance class 0) and among individuals separated across a range of distances from 0 to 100 km evaluated at 5 km increments. Our data was tested against the null hypothesis of randomly distributed genotypes, with 999 permutations and 999 bootstrap replicates.

### Simulations of population genetic structure

Next, we simulated genetic differentiation under a range of dispersal scenarios and compared these results with our microsatellite data. To do this, we used IBDSim v.2 [69] to simulate genotypic data for multiple unlinked loci under a general isolation-by-distance model. IBDSim is based on a backward-in-time coalescent method that enables the generation of large data sets using complex demographic scenarios. For our simulations, we constructed a 100 km × 0.5 km matrix that was representative of the entire intertidal area between the two most distant sample sites on Guam (Pago to Adelup Point; Fig 2i). The distance of these sites set the outer spatial limits of our matrix. The matrix was composed of 50,000 grid squares with each square 10 m × 10 m in area. In each simulation, we populated the matrix with 10, 20, 50, 100, 500 or 1000 larval fish per grid square, which corresponds to densities of 0.1, 0.2, 0.5, 1, 5 and 10 larvae per m^2^, respectively. These densities were chosen as input parameters based on empirical estimates of the total adult density of *A*. *arnoldorum* obtained for five of the six sampling locations by another study [44] conducted a month after the collection of tissues for the current study. The empirical estimates ranged from 1.3 to 9.3 individuals per m^2^ (average 4.8/m^2^). Our simulations therefore provide an assessment of genetic differentiation across a reasonable range of population densities (although we acknowledge that the density of larvae and adults might differ in reality).

For each simulated population density, we used input parameters that closely matched those of our empirical dataset. These included 17 microsatellite loci under a strict stepwise mutation model (SMM; [70]) using a mean mutation rate of 0.001 [71]. To this we applied six different dispersal distributions (named in the IBDSim Manual as ‘0’, ‘2’, ‘3’, ‘6’, ‘7’, and ‘9’; [69]) to model various degrees of dispersal around the inter-tidal matrix. These dispersal distributions have similar total emigration rates and mostly differ in their ‘shape of dispersal’ characterised by the mean squared parent-offspring dispersal distances (σ^2^). For our simulated matrices representing a range of dispersal scenarios, the default values defined by IBDSim for dispersal distributions correspond to mean squared parent-offspring dispersal distances of 10 m, 40 m, 100 m 200 m, 1000 m. These distances can be interpreted as the average squared axial distance that offspring of a common ancestor will become separated per generation [72, 73]. These mean squared parent-offspring dispersal distances are paired with different combinations of *M* and *n* that control the maximum dispersal rate per generation and kurtosis (a measure of shape) of the dispersal distribution per generation respectively (see IBDSim Manual; [69]). For each simulation, the maximum possible dispersal distance was capped at 100 km (i.e., to the size of the largest distance possible in the matrix), which is also a realistic value assuming Lagrangian dispersal [43] and a one month larval phase (Platt and Ord, unpublished data). The boundary of the matrix was set to ‘absorbing’ in which individuals that emigrate out of the lattice are lost (i.e. swept out to sea). All simulations used a truncated Pareto distribution (e.g. [74]) that allows for high dispersal rates as expected in the marine environment and is characterized by high kurtosis, which is often observed in biologically realistically functions [75, 76]. This distribution assumes a high probability of dispersal per generation over a relatively small distance, and decreasing probability for higher distances. We sampled fish from the simulated lattice from 100 evenly distributed locations (each population 1 km apart). Ten replicate analyses were conducted for each simulation combination. We then used Genepop version 4.0.10 to calculate global *F*_ST_ between the simulated populations and compared this with the global *F*_ST_ from our empirical data. The simulated *F*_*ST*_ values were approximately normally distributed and we subsequently used the standard deviation of *F*_*ST*_ values to calculate where 99% of values would theoretically lie in a normal distribution (i.e z = ±2.576) to provide a “99% percentile” for *F*_ST_ values at each density.

### Meta-analysis of population structure

To place our microsatellite data set within the broader and generalised context of population genetic structure in fish we examined the slope of *F*_ST_ over geographic distance in marine fish from published studies. This enabled us to estimate the rate at which genetic differentiation accumulates as a function of geographic distance. To collect these data, a systematic literature search was conducted in Web of Science®. Titles, abstracts and keywords of all articles published between 2006 and 2011were searched for using the terms: ‘phylogeography*’, ‘population genetic structure*’, ‘population genetic*’ and ‘landscape genetics*’. Of the 612 articles pertaining to fish, 66 focused on marine fish, employed microsatellite markers, compared more than three populations, provided usable geographic information and measured pairwise *F*_ST_ (Fig 1)

For each of these studies, we measured the Euclidian distance between the two closest and the two furthest populations. We then recorded the pairwise *F*_ST_ for the population comparisons and calculated the slope for each study as:

$$\beta =\frac{\Delta F_{ST}}{\Delta Distance},$$ eq 1

Where Δ*F*_ST_ is the difference between the pairwise *F*_ST_ of the furthest populations and the pairwise *F*_ST_ of the closest populations, and ΔDistance is the difference between the pairwise distance in km of the furthest populations and the pairwise distance in km of the closest populations. With only two data points collected per study, linearity of the relationship between *F*_ST_ and distance could not be tested for specifically. However, linearity is commonly assumed (i.e. Mantel test for IBD) and our analysis also relies on this assumption. We calculated the average number of individuals per population sampled per study to provide an approximate measure of precision that was then used to obtain a weighted average *β* for each species. Unweighted averages were also assessed but these gave very similar results and did not change any of the conclusions. Additionally, we recorded for each study whether or not spatial population structure was present (statistically significant pairwise population *F*_ST_ values), and where tested by the authors, whether or not there was IBD (statistically significant correlation between geographic distance and *F*_ST_) or panmixia. This enabled us to test for any association between our measure of *β* and IBD (or lack there of) identified by the authors. For these analyses, species averages were not used to allow for comparison across studies.

The slope estimates computed from Eq 1 provided a standardized measure of the extent to which geographic distance influences *F*_ST_. This was used instead of simply comparing “raw” *F*_ST_ values by distance because the magnitude of individual *F*_ST_ values will differ depending on the number of alleles within each sub-population examined by a study [27, 29, 77]. Computing a difference score between *F*_*ST*_ values estimated for the furthest and nearest population reported by a study helps control for this potential bias among studies since we are comparing the rate at which *F*_*ST*_ accumulates with distance across studies rather than raw *F*_*ST*_ values. Moreover, the geographic distance at which the maximum pairwise *F*_ST_ occurs has been documented to be highly variable (see [78]), and thus a measure of slope was a comparable metric between studies.

Where data were collected for the same species over multiple studies, the average slope between studies, weighted by the average number of individuals per populations in each sample, was calculated. This reduced our sample size from 66 studies to 58 distinct species. The confidence interval for the slope was then estimated using a bootstrapping percentile procedure in R version 2.15.0 (R Development Core Team, 2012). Bootstrapping was weighted by average sample size (NB: unweighted bootstrapping gave very similar results). The slope for *A*. *arnoldorum* was calculated with a Mantel test on microsatellite data. Due to the non-independence of pairwise comparisons and sample size (six populations), no confidence intervals for the *A*. *arnoldorum* estimate were calculated.

We also used our meta-analysis data to estimate the geographic distances necessary to achieve a range of genetic differentiation values for marine fish more generally. For each species, the linear line connecting *F*_ST_ between the closest and furthest pairwise populations on a plot of distance (x-axis) by *F*_ST_ (y-axis) was calculated (the slope of this line is calculated in Eq 1). The line for each species was then extrapolated so that the necessary pairwise geographic distances needed to achieve any given *F*_ST_ value could be estimated. Therefore, distance (*d*) was calculated as:

$$d=\frac{F_{ST}-\alpha }{\beta }$$ eq 2

Where *α* is the intercept of the extrapolated line, and *β* is the slope of this line (calculated in Eq 1). For each distance estimated, the median (50%) distance over all species was bootstrapped to estimate confidence intervals with the percentile procedure in R [79].

## Results

### Sequence analysis and demographic history of *Alticus arnoldorum*

We aligned the entire 842 base pairs (bp) of ATPase 6 and 8 for 120 individuals including the start and stop codons for each gene. These were composed of 29 unique haplotypes, defined by 25 variable characters of which seven were parsimony-informative. Summary statistics for the mitochondrial data are shown in Table 1. Based on the haplotype network for which no unresolved loops formed (Fig 2iii) there is little association between sampling location and haplotype, such that the four most common haplotypes (1–4) are sampled in nearly equal proportions from each site. Nonetheless, at each sample location, there are up to four unique and recently derived haplotypes present in the network.

Analyses of demographic trends in *A*. *arnoldorum* on Guam suggest a recent population size increase that may have occurred during the late Pleistocene. While analyses based on a single molecular clock must be interpreted with caution, BSP analysis indicated that *A*. *arnoldorum* population size increased on Guam approximately 20 thousand years ago (S1 Fig). Consistent with this finding was Fu’s test of selection/demographic change that gave a significant and large negative *F*_S_ (-26.398, *P* = < 0.01) a result also indicative of demographic expansion or directional selection. For the mismatch analysis however, our data deviated significantly from the model expected under demographic expansion (Sum of squared deviation = 0.0126, *P* = 0.0121; Harpendings Raggedness index = 0.1200, *P* = 0.0003). However, the distribution of the observed number of pairwise differences was unimodal in distribution, which is expected of populations experiencing demographic expansion [54].

### Microsatellites

At the 17 polymorphic loci, there were an average of 16 alleles per locus (ranging from 4 to 31). Within each sampling location, the average H_O_ ranged from 0.645 (TS) to 0.703 (AP). Observed and expected HWE values and their associated *P*- values for each locus within each sampling location are shown in S2 Table. Within each population, there was significant deviation from Hardy-Weinberg equilibrium (HWE) at some loci after sequential Bonferonni correction, however only one locus AR06 consistently deviated from HWE and was subsequently removed from analyses of population structure. In nearly every population, heterozygosity was lower than expected for most loci, although this deficit was not necessarily statistically significant. This result may be the product of either null alleles or inbreeding. Results from Microchecker found that there does not appear to be any scoring error or allele dropout, but at approximately half the loci, null alleles may account for the homozygosity excess observed in our data. Further, the multilocus “null free” average inbreeding coefficient (*F*_IS_) as calculated by INEst ranged from 0.004 to 0.006 and was much lower than *F*_IS_ derived using 1-H_O_/H_E_ (S2 Table). This suggests that the heterozygote deficit observed in this dataset can be better accounted for by null alleles than by inbreeding depression. Thus, to check that the presence of null alleles was not biasing our results, we ran the same analyses for the data set excluding the markers highlighted using Microchecker.

### Population genetic structure

Based on both the mitochondrial and microsatellite data, there appears to be very little population genetic structure in *A*. *arnoldorum* on Guam. For the microsatellite dataset, overall genetic differentiation (*F*_ST_) was very low (0.0043) and changed little with the removal of the loci with null alleles (0.0053). Analysis of pair-wise population structure based on mtDNA Φ_ST_ was very low and non-significant for all population comparisons (S3 Table) and, correspondingly, there was no relationship between geographic distance, (Euclidian or coastal), and Φ_ST_.

For the total microsatellite dataset, we have chosen to report only *F*_ST_ since both G’_ST_est_ and D_est_ were correlated with *F*_ST_ (*F*_ST_ vs G’_ST_est,_ R = 0.72, P<0.01; *F*_ST_ vs D_est,_ R = 0.66, P<0.01). Pair-wise population structure based on *F*_ST_ was very low (ranging from 0.0008–0.0095) yet a statistical effect was discernible after Bonferroni correction in five of the 21 pairwise comparisons (S4 Table), while mutual information (also called ^*S*^*H*_*UA*_) was also very low and ranged from 0.021–0.032 and had no statistically distinguishable effects in any pairwise comparison. In a similar manner to mtDNA, there was no relationship between geographic distance (Euclidian or coastal) and *F*_ST_. Generally, the same pattern of significant pairwise population structure (*F*_ST_) was observed across the matrix following the removal of the loci that had putatively null alleles, with the exception of two pairwise comparisons (S4 Table). Despite this discordance, pairwise F_ST_ was low with or without null alleles and indicated little if any population genetic structure among the sampled populations. This was corroborated by results from Structure that showed the highest mean estimated logarithm of likelihood for K to be 1, which also exhibited the smallest standard deviation. Following the Evanno et al. [68] method, the distribution of ∆K supported an optimal number of two clusters, but individuals did not cluster in any meaningful way in respect to sample location (S2 Fig). Thus, our data is consistent with a pattern of one genetic cluster.

**Fig 4:**
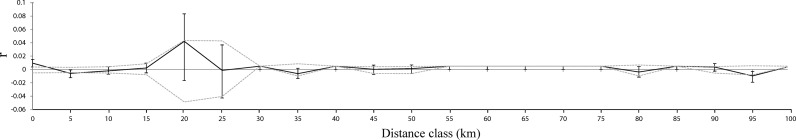
Spatial autocorrelation analysis. Based on 204 *Alticus arnoldorum* samples for microsatellite data excluding putative null alleles. Autocorrelation *r* values (black line) are presented in relation to the 95% confidence belt (dotted lines). Error bars at each distance class represent the confidence interval around the observed value of *r* based on 999 bootstrap permutations of the data. The probability values for a one-tailed test for positive autocorrelation, together with upper and lower bounds for the confidence intervals and bootstrap re-sampling are in S5 Table.

From microsatellite spatial autocorrelation analysis, there was a small, but statistically distinguishable effect of positive spatial structure (greater than random genetic similarity) is present between pairs of individuals from the same sampling site, regardless of whether or not the putative null alleles were excluded or included in the analysis (total data set r = 0.007, *P* = 0.01; without null alleles r = 0.009, *P* = 0.01) (Fig 4; S5 Table). However, similar to *F*_ST_ and Structure analyses, there was no significant spatial autocorrelation among individuals sampled in different localities with the exception of the 20 km distance class i.e. the probability was greater than 5% of randomly achieving an individual *r* value greater than or equal to the observed r value for all distance classes except 20 km (S5 Table). At 20 km there was a sharp spike in autocorrelation signal (total data set r = 0.011, *P* = 0.19; without null alleles r = 0.042, *P* = 0.04) indicating possible greater than random genetic similarity. However, given the large increase in variance around *r* within this distance class, this result should be interpreted with caution.

### Simulations of genetic structure

**Fig 5:**
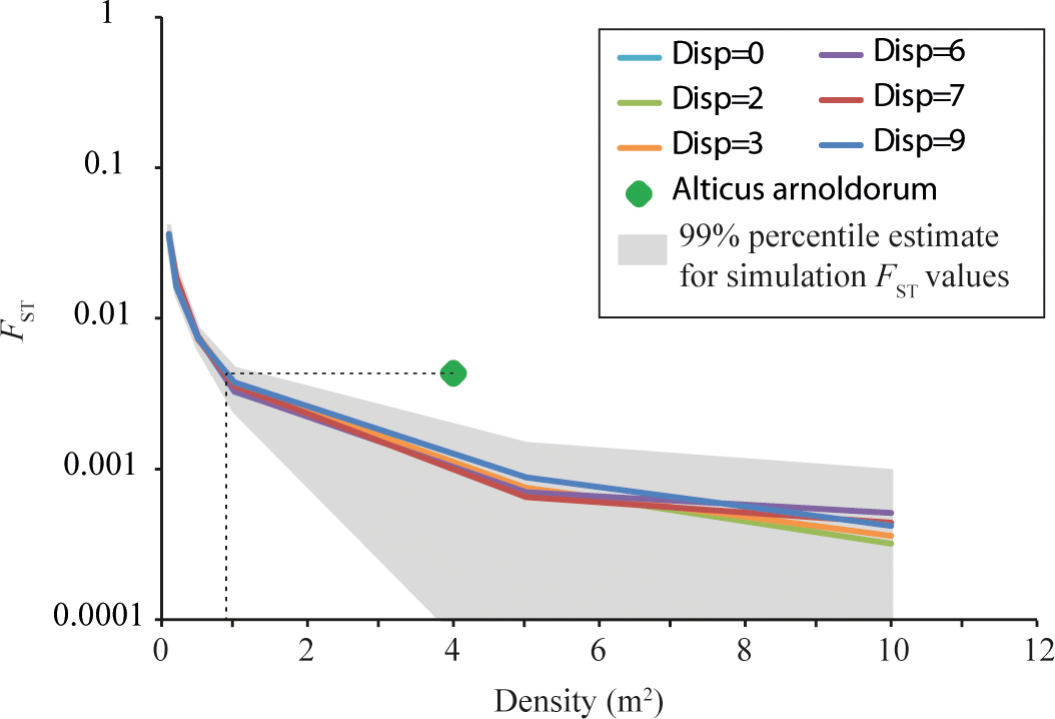
Results of simulation analyses of *A*. *arnoldorum* around Guam illustrating the relationship between density (m^3^) and *F*_ST_. Each coloured line represents a different dispersal scenario employed in simulations and the green diamond represents the global empirical *F*_ST_ for *A*. *arnolodorum*. Dispersal distribution ‘0’, ‘2’, ‘3’, ‘6’, ‘7’, and ‘9’ correspond to mean squared parent-offspring dispersal distances of 10 m, 40 m, 100 m 200 m, 1000 m respectively.

In each of our computer simulations, *F*_ST_ was more dependent on grid population density than dispersal scenario (Fig 5): global *F*_ST_ decreased as grid density increased. In other words, as the available habitat became increasingly populated, the increase in effective population size ensured a greater likelihood of gene flow among populations. To obtain a global *F*_ST_ comparable to that computed empirically for *A*. *anolodorum* (0.0043), our simulations suggest a population density just below one individual m^-2^ (Fig 5). The average population density of adult *A*. *arnoldorum* is more likely closer to five individuals m^-2^ [44], which would be consistent with a simulated *F*_ST_ below 0.001 (Fig 5). This could reflect a number of things: (i) that the overall dispersal rate of *A*. *arnoldorum* was lower than those simulated; (ii) the density of larval fish was lower than settled adult populations; or (iii) that the distribution of individuals was fragmented around the circumference of Guam.

### Meta-analysis

**Fig 6:**
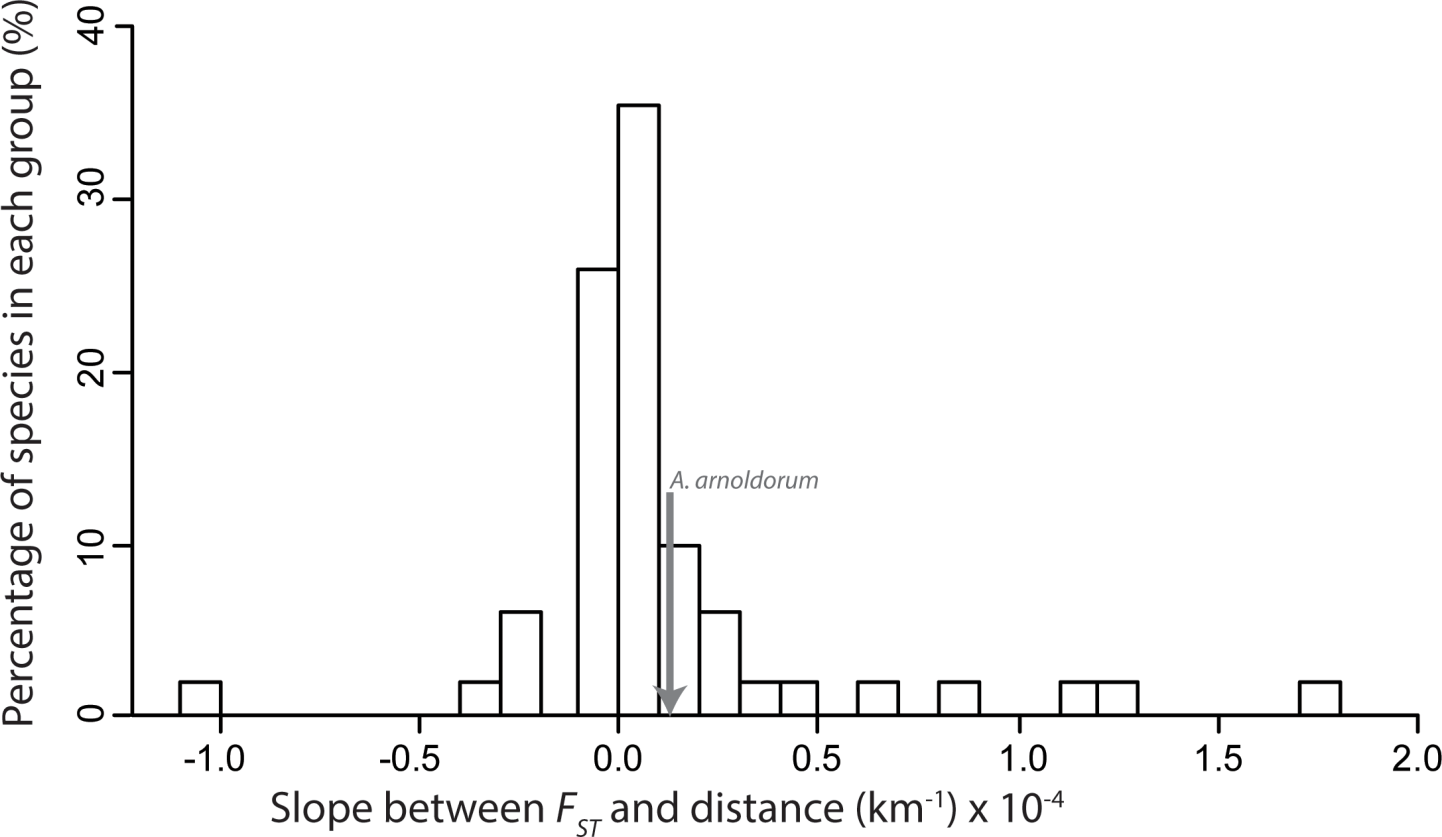
Histogram of meta-analysis data. Showing 90% of the slopes between *F*_*ST*_ and distance (km). 10% of data has been excluded for visual clarity (2% below and 8% above histogram range). Excluded outlier values are: -4.9×10^−3^; -3.2×10^−3^; 3.2×10^−4^;3.8×10^−4^; 6.0×10^−3^;1.3×10^−3^; and 1.7×10^−3^.

Using the meta-analysis to calculate a generalised trend of *F*_ST_ slope over geographic distance (*β*) not surprisingly we found considerable variation across the published studies: slopes ranged as low as -0.0049 km^-1^ (negative slopes are consistent with recruitment to natal sites) to as high as 0.0017 km^-1^ (suggesting possible active dispersal from natal sites). However, the majority of *β* values were clustered close to zero with the interquartile range lying between -6.0 x 10^-7^km^-1^ and 2.1 x 10^-5^km^-1^ (Fig 6; Table 2). Reef-associated tropical species were also analysed separately to check for any correlation between reef lifestyle and *β* yet their median *β* was 4.04 x 10^-6^km^-1^ and still within the interquartile range for all fish species. This was also the case for *A*. *arnoldorum* that was computed to have a slope of 0.12 x 10^-4^km^-1^ and found to have no IBD using Mantel tests (see ‘Population genetic structure‘ above’).

**Table 2:** Rates of *F*_ST_ over geographic distance (*β*) collected in the meta-analysis of marine fish. For the combined data set, the number of studies equals the number of species (slopes averaged across studies; see Materials and methods).

	Combined Data	Studies identifying IBD	Studies identifying no IBD (and Panmixia)	Studies in which IBD was not tested
**Number of studies**	58	16	25	25
**Median *β* (km**^**-1**^**) × 10**^**−4**^ **(bootstrap 95% C.I.)**	0.016 (0.0044 to 0.096)	0.19 (0.011 to 1.14)	0.015 (0 to 0.098)	0.015 (0 to 0.063)
**Interquartile range *β* (km**^**-1**^**) × 10**^**−4**^	-0.006 to 0.21	0.009 to 1.19	-0.022 to 0.15	-0.0084 to 0.09
**Minimum *β* (km**^**-1**^**)**	-0.0049	-2.0 × 10^−6^	-1.01 × 10^−4^	-0.0061
**Maximum *β* (km**^**-1**^**)**	0.0017	0.0013	2.7 × 10^−4^	0.0017
**P-value for median *β* difference from zero**	0.004	0.003	0.21	0.28

From the 66 studies included in our meta-analysis, 74% were found to have a positive *β* as measured using Eq 1 while the remaining 26% were found to have a negative *β* (Fig 6; Table 2). Of these 66 studies, 20% reported no spatial genetic structure (no significant pairwise *F*_ST_ comparisons), 15% reported little to no spatial genetic structure (few significant pairwise *F*_ST_ comparisons), while 65% reported spatial genetic structure (the majority of pairwise *F*_ST_ comparisons were significant) (S6 Table). The most common explanations for spatial genetic structure included biogeographic history, habitat boundaries and oceanographic patterns. Only 37 of the 66 studies specifically tested for IBD (using a Mantel test or similar), and of these, just 16 studies reported a significant correlation between geographic distance and *F*_ST_. Consistent with this, we found that the median *β* was higher in studies that report IBD (median *β* = 0.19; CI = 0.011–1.14) compared to studies that found no evidence of IBD (median *β* = 0.015; CI = 0.0–0.98; Table 2), although this latter result was marginally non-significant in two-tailed tests (*P* = 0.08 IBD vs. No IBD, *P* = 0.11 IBD vs. No IBD and Panmixia). However, the median *β* in studies that identified IBD was significantly different from zero (*P* = 0.003; Table 2), unlike studies that did not find IBD in which *β* was non-significantly different from zero (Table 2). These were important results as they confirmed that our two-point estimate of *β* (Eq 1) was generally consistent with the overall spatial genetic structure reported in each study.

**Fig 7:**
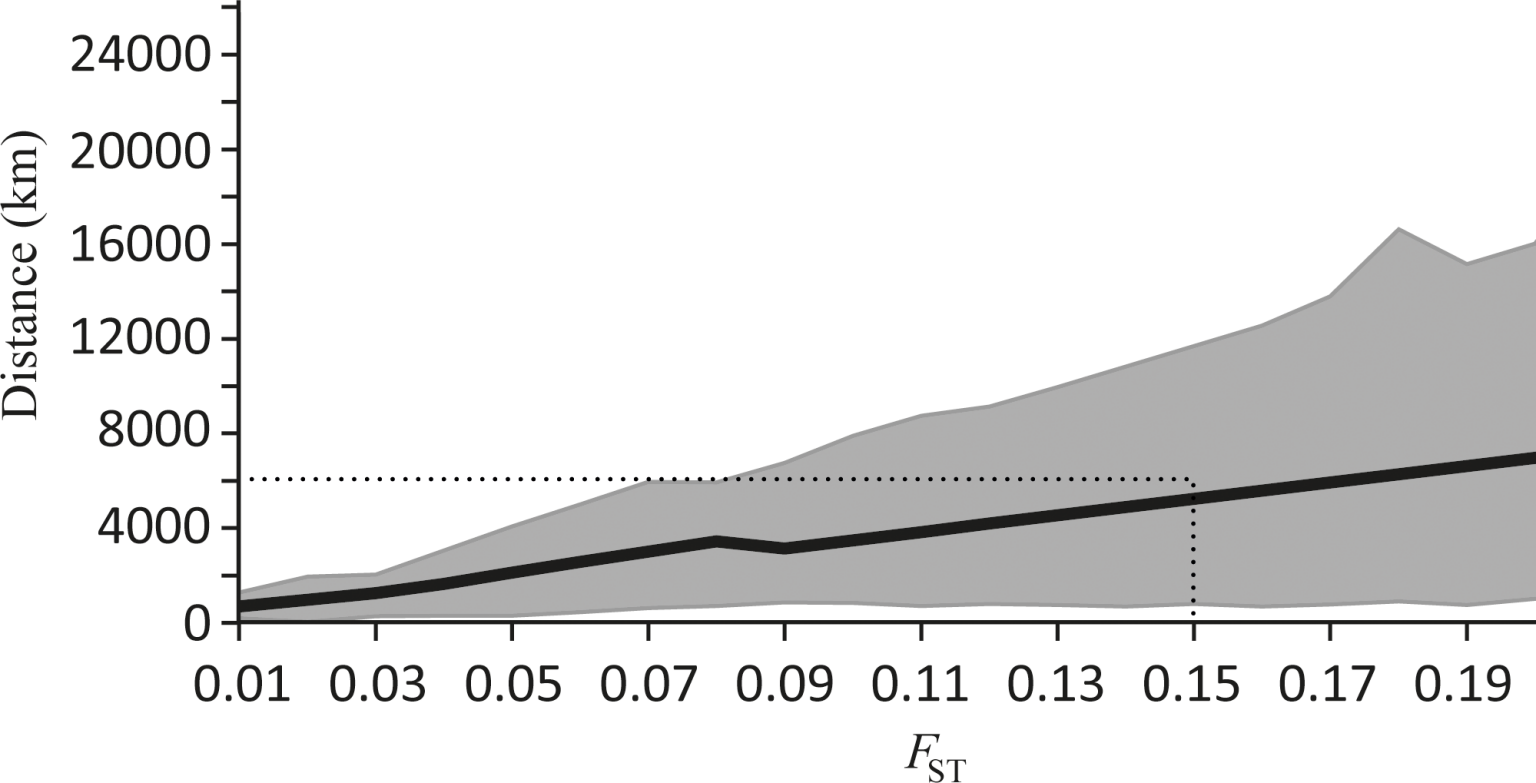
Geographic distances (km) expected between populations of marine fish with increasing *F*_ST_ based on meta-analysis data. The black line represents the median distance expected for an *F*_*ST*_ value and is presented in relation to the 95% confidence intervals (grey dotted line) (S7 Table). An *F*_ST_ of 0.15 has been marked on the graph as it generally is considered to be significant [2].

By extrapolating the complete meta-analysis data set and assuming that the relationship between *F*_ST_ and distance is linear in our two point per species data set (or at least locally linear for small *F*_ST_ values), we predicted the geographic distance at which a given *F*_ST_ is likely to be observed (Fig 7; S7 Table). This showed that, in general, *F*_ST_ accumulates slowly across vast oceanic distances for fish, although this result should be interpreted with some degree of caution due to the wide confidence intervals associated with our *β* median estimates. Nonetheless, to obtain a level of genetic isolation generally considered to be important for evolution, i.e. *F*_ST_ = 0.15 [2], the data suggests that the minimum distance between populations for the “median fish” would need to be at least 5242 km (95% C.I. 810–11692 km; Fig 7). Despite the considerable variance in *β* among studies, even the lower confidence interval of this estimate suggests a degree of connectivity that is much higher than generally appreciated in the literature (e.g., connectivity in marine fishes is likely to be much higher than 300 km; see Introduction). Publication bias could not be measured meaningfully for this data set due to the association between sample size and effect size. Nonetheless, publication bias in this context (i.e. an underrepresentation of published studies that found no spatial genetic structure—translated to *F*_ST_ slopes of zero in our meta-analysis) would result in our overall estimate of *F*_ST_ slope with distance presented in the article to be greater than it should be. This would mean that *F*_ST_ accumulates even more slowly across vast oceanic distances already supporting our conclusion (i.e. no publication bias should have no impact on our qualitative result).

## Discussion

By comparing empirical data of a species whose ecology effectively eliminates adult dispersal (the land fish, *Alticus arnoldorum*) to biologically informed simulations and a large meta-analysis of published literature, we provide a broad estimate of the patterns and spatial scale of genetic connectivity at sea. Our comparison was framed around three alternative scenarios of how the behaviour of pelagic larvae might impact genetic connectivity among marine populations. Our results suggest that a scenario involving both passive and active larval dispersal explains the extensive connectivity among populations of *A*. *arnoldorum* (Scenario 3 in Fig 2), and possibly many published studies on marine fish more generally (e.g. [13, 80–82]). This implies that the high genetic connectivity often assumed to occur in marine environments [9–12] and confirmed by the results of our meta-analysis, can be maintained by a pelagic larval phase even when adult populations are separated from one another by ecological barriers. Moreover, our meta-analysis provides a broad estimate on the spatial scale necessary for evolutionary meaningful genetic differentiation to occur among populations of marine fish. This result has important implications for how we make generalisations about speciation in marine environments. In other words, understanding the rate at which genetic differentiation accumulates in the sea provides us with a means to estimate the effect of geographic distance on speciation for fish.

### Population genetics and demographic history of *Alticus arnoldorum*

While our results clearly showed an absence of spatial genetic structuring and IBD in both microsatellite and mitochondrial DNA among sampled sites of *A*. *arnoldorum* around Guam (Fig 2; S2 Fig; S3 and S4 Tables), some “chaotic genetic patchiness” was nevertheless detected (Fig 4). The rate of IBD in *A*. *arnoldorum* also fell well within the interquartile range of *β* for published studies for marine fish (Fig 6). Given the ecological isolation of adult *A*. *arnoldorum* populations on land, this strongly indicates dispersal among populations via pelagic larvae. However, the absence of strong spatial genetic structure might also reflect one of the following: high effective population sizes, or a lack of sufficient time for genetic drift to have accumulated between isolated populations. Given the demographic expansion or colonization of Guam by *A*. *arnoldorum* (Mismatch analysis, BSP: S1 Fig) we can calculate the expected time (*T*) for a pair of populations to reach 50% of the drift-dispersal equilibrium *F*_ST_ using the following equation [83]:

$$T=\frac{\text{ln}\left(0.5\right)}{\text{ln}\{[{\left(1-m\right)}^{2}\times \left[1-\frac{1}{2\text{Ne}}\right]\}}$$ eq 3

If we assume a maximum larval density for *A*. *arnoldorum* of five larvae m^-2^ (e.g. [44]), that dispersal between a pair of populations (*m*) is 1% per generation and that the effective population size (Ne) is 10% of the maximum population density [2] then *T* is approximately 23 generations. This would be well within the timescale predicted using Bayesian Skyline Plot analysis (S1 Fig). It seems more likely then that the genetic homogeneity observed on Guam is the product of high larval-based gene flow and high effective population sizes. Both high larval-based gene flow and high effective population sizes appear to independently contribute to genetic homogeneity in many marine taxa [9, 24, 84].

The patterns of ocean circulation around Guam are generally both spatially and temporally variable with an overall flow that fluctuates from westward to northward at speeds of 0.1–0.2 ms^-1^ [85]. At the lowest flow speed of 0.1 ms^-1^, it is possible for a passively drifting particle to travel ~242 km during the time of the average pelagic larval phase of an *A*. *arnoldorum* (one month; Platt and Ord, unpublished data). This distance is less than the 300 km estimated under a Lagrangian dispersal model for the same time frame [43] yet still further than the maximum coastal distance between any two of our sample sites (91 km). It therefore seems that Guam represents a single genetic population of *A*. *arnoldorum* despite adult populations being ecologically isolated from one another. This is common in coral reef fish [5, 81, 86, 87], where significant genetic structuring can often only be detected at the largest of spatial scales [12, 88–92].

Despite the general lack of genetic structuring and IBD among *A*. *arnoldorum* populations (Fig 2; S2 Fig; S3 and S4 Tables), there was still evidence for some positive spatial structure within short distances (greater than random genetic similarity: Fig 4). This fine scale patchiness with broad scale genetic homogeneity, or “genetic patchiness” (Scenario 3; [45, 46]), is what differentiates our results from the passive larval dispersal model of Scenario 2 (Fig 3I). Chaotic genetic patchiness is common in the marine environment [93] and can result from factors such as active dispersal, natural selection acting before or after settlement, population recruitment or cohesion of larvae which are then diluted in the long-term by gene flow and dispersal (e.g. [37, 94–96] or temporal changes in the sources of larvae that settle to a given location. For example, numerous studies have reported reef fish larvae with highly directional swimming, which gives them the capacity to minimize the influence of ambient currents and enables them to settle in their natal reef habitat [13, 81–82]. Such directed dispersal by larvae can vary the genetic composition of populations independently of geographic distance [45, 46, 78].

Our simulations of genotypic data (Fig 5) were also consistent with scenario 3. The empirical estimate of genetic differentiation among populations of *A*. *arnoldorum* was always higher than those simulated which again implies chaotic genetic patchiness (Fig 4).

### Genetic connectivity in the marine environment

Despite many studies detailing species-specific relationships between genetic connectivity and spatial population structure in the marine environment, there is still limited information about the prevailing patterns with respect to spatial gradients. In general, dispersal estimates based on IBD regressions (Mantel tests or similar) have been shown to reflect direct estimates of dispersal in mammals (e.g. [97]), reptiles (e.g. [98]), insects (e.g. [99]) and plants (e.g. [100]). Yet, whether or not IBD reflects the typical spatial organisation of marine fish is debateable (e.g. [78]). The results from our meta-analysis provide the first examination of these trends and we estimate the generalised spatial scale at which population genetic structure accumulates over distance for a fish in the ocean. Although our results are a generalisation and do not account for nuanced species specific life history traits, the outcome of our meta-analysis is still an important step towards understanding the scope of connectivity in the marine environment. Arguably, quantifying and understanding the relationship between connectivity and geographic scale is recognised as one of the most critical issues in marine ecology to date [18]. Put simply, spatial information of this sort could be used to determine the scale over which populations of marine fish may interact, the scale over which fisheries should be managed, and the way in which marine protected networks should be designed and implemented [18].

Overall, our meta-analysis agrees with general assumptions about marine dispersal and suggests that connectivity is high and genetic differentiation with geographic isolation appears to accumulate slowly at sea for fish in general. For the majority of studies, *β* (the rate at which genetic differentiation accumulates with distance: Eq 1) clustered closely to zero (Fig 6; Table 2; S6 Table). This result may be consistent with the assumption that there are few obvious physical barriers in the ocean and that pelagic larval dispersal can lead to high genetic connectivity over large geographic distances. Moreover, this appears to occur among adult populations that may be otherwise isolated from one another by ecological barriers. Indeed, *β* in *A*. *arnoldorum* sits within the interquartile range of published studies (Fig 6), yet it is also a species where adult populations are ecologically isolated from one another. The implication of this result is that marine fish populations may still be isolated as adults but otherwise connected by larval dispersers that cross or circumvent the ecological barriers separating adult populations. Our finding that larval dispersal in *A*. *arnoldorum* is likely a combination of passive and active dispersal (prediction 3; (Fig 3i and 3ii)) is consistent with the well established notion that at least some degree of larval dispersal either active, passive or a combination of both (i.e. >150 km [101]) is also widespread in marine fish (e.g. larval coral reef fishes; [81, 101]) and this can translate into genetic connectivity that is vast over large spatial scales for many species.

The prevailing spatial pattern of genetic connectivity in marine fishes does not seem to be IBD. More than 60% of the studies included in our meta-analysis reporting spatial genetic structure, few of these (16 studies) actually identified IBD (S6 Table). In the remaining cases, various explanations were reported to account for the spatial genetic structure, including biogeographic history, habitat boundaries, oceanographic patterns and demographic history to name a few (see S6 Table for a complete listing). Indeed, stepping stone models of dispersal as explanations for spatial genetic structure were rarely evoked. This result was consistent with a recent survey of vertebrates, invertebrates and plants that found that IBD was only identified in 20% of studies [102]. Thus, when the median *β* was calculated separately for studies exhibiting IBD compared to those that did not (or did not specifically test for it), not surprisingly, we found that the *β* was considerably higher and significantly different to zero in studies that found IBD compared to those that did not (Table 2). This result suggests that populations exhibiting a stepping stone model of dispersal will accumulate genetic structure more rapidly over distance compared to those that do not, even when equal amounts of spatial genetic structure are present.

The variety of causes likely to account for the spatial genetic structure observed in each study (i.e. species specific life history traits) presumably underlies the considerable variance in *β* in our meta-analysis (Fig 6; Table 2). This was evident in the wide confidence intervals associated with our prediction of the extent to which *F*_ST_ will increase with geographic distance (Fig 7). Indeed, this is a limitation of pooling data across species to obtain a highly generalised picture of dispersal. Nevertheless, we can tentatively estimate the spatial scale at which appreciable genetic differentiation (based on microsatellite markers) might accumulate between populations for a median marine fish (e.g. *F*_ST_ = 0.15; [2]). Our meta-data suggest that populations would need to be approximately 5,000 km apart, with a lower and upper estimate of 810 and 11,692 km, respectively (Fig 7). This result must be interpreted with caution given the assumption of linearity applied here and the scale over which most studies are conducted (hundreds of kilometres). Thus, the extrapolation of the relationship to thousands of kilometres may indeed limit the accuracy of our result. Moreover, given the broad confidence intervals of this median estimate, it is important to remember that strong population structure can occur on the scale of tens of kilometres (e.g. [103]), and population structure need not necessarily be present over 5,000 km (e.g. [104]). However, despite applying an assumption of linearity here and the variability on a case by case basis, the overall pattern is consistent with the notion that particularly vast distances are necessary to achieve appreciable genetic structure among populations, and this probably reflects the high dispersal capacity of larvae and the general absence of physical barriers to this mode of dispersal in the marine environment.

It is also important to recognise that low *F*_ST_ values are generally expected for highly heterozygous markers such as microsatellites [28], which can also limit the resolution of weak genetic structure–a characteristic typical of marine organisms [105]. This particular characteristic of our data would bias the meta-analysis to a shallower slope and thus a higher inferred connectivity distance for any given pairwise *F*_ST_ comparison. In addition, frequently used measures of genetic connectivity including *F*_ST_ may also over-estimate population connectivity (e.g. demographic processes, also known as “demographic connectivity”). This is because it takes only a few migrants between populations per generation to prevent the accumulation of appreciable genetic differentiation as presumed by *F*_ST_ [106]. Indeed, infrequent stochastic dispersal events may be maintaining genetic exchange across vast distances between otherwise isolated populations [24, 106] and as a result, long distance passive larval dispersal may actually be rare and have little demographic input [24, 25]. Taken together, estimates of connectivity based on microsatellite data should be interpreted as outer limits for which other measures of connectivity (e.g. the movement of individuals between populations that is of demographic significance) will generally not exceed.

With this in mind, the degree to which populations are connected based on our meta-data still has some potentially important ramifications for understanding how species respond to selection and adapt to environmental change [2]. Even rare genetic exchanges between populations separated by large spatial scales (i.e., resulting in high genetic connectivity) could lessen adaptive change to local environments as well as impact the overall likelihood of speciation by homogenizing populations genetically. Conversely, despite this connectivity among populations, the number of dispersing individuals may not be enough to rescue a threatened population from local extinction (e.g. those heavily harvested; [9]).

### Conclusion

There can be certain caveats associated with making generalisations about connectivity based on *F*_ST_ (e.g. inflation of connectivity estimates [28], non-adherence of data to stepping stone model [24, 78, 106] and amalgamation of species specific life history traits). However, by employing the combined approach of empirical data, simulations and a meta-analysis we have evaluated the extent to which pelagic larval dispersal in fish likely impacts genetic connectivity among populations that may otherwise be isolated from each other. Using the unusual land fish, *A*. *arnoldorum*, as a model, and comparing these results with a meta-analysis, we have been able to assess general patterns of spatial genetic structure in marine fish and provide a broad estimate of the spatial scale of genetic connectivity that would be impossible using a single approach [107]. This estimate of genetic connectivity is useful for understanding both speciation as well as the conservation implications of spatially oriented resource management in the marine environment. In fact, measures of genetic connectivity such as *F*_ST_ are being readily incorporated into the design of marine protected areas and reserves e.g. [8, 16, 18, 21, 24,108]. With major declines observed in fishery stocks, the accelerated degradation of coastal habitat and climate change, understanding the complexity of connectivity in marine organisms, including genetic connectivity, has never been more critical for the conservation and management of marine environments. Indeed, understanding genetic connectivity in this context will ultimately assist us to diagnose the resilience of populations and species in our marine habitats.

## Supporting Information

S1 Fig PRISMA 2009 Checklist. (DOC)

S2 Fig Bayesian skyline plot derived from the ATPase 6 and 8 sequences (*n* = 120) showing the effective population size as a function of time. The thick black line is the median estimate of the log_10_ of the effective population size, and the thin grey lines are the 95% higher posterior density.
(TIF)

S3 Fig STRUCTURE results based on 204 *Alticus arnoldorum* samples for microsatellite data excluding putatively null alleles: (i) K = 2 after Evanno et al. [68] and (ii) K = 6. Individuals are grouped by sampling location and each individual is represented by one vertical line broken into K coloured segments, with the lengths being proportional to the K inferred cluster.
(TIF)

S1 Table Characterisation of the 17 polymorphic microsatellite loci for *Alticus arnoldorum* (N = 204) and multiplex panel design. Types of fluorescence used to label forward primers are indicated with the primer sequence (FAM, NED, PET, VIC). N_A_, number of alleles.
(DOCX)

S2 Table Descriptive statistics and diversity indices for each population per locus. *N*_a_, number of alleles per locus; A_r_, allelic richness; *F*_IS,_ Wrights inbreeding coefficient; HW Obs, Hardy-Weinberg observed heterozygosity; HW Exp. Hardy-Weinberg expected heterozygosity; and HW p-value, Hardy-Weinberg P-value; *, significant after sequential Bonferonni correction.
(DOCX)

S3 Table Pairwise Φ_ST_ comparisons for the 7 sampled populations of *Alticus arnoldorum*. No comparisons were significantly different.
(DOCX)

S4 Table Pairwise F_ST_ comparisons for the 7 sampled populations of *Alticus arnoldorum*, (i) total data set, (ii) data set excluding null alleles (*P≤0.05 after bonferroni correction). (DOCX)

S5 Table Spatial Autocorrelation analysis for the microsatellite data set excluding putative null alleles. The number of pairwise comparisons, *N*, correlation, r, upper U and lower L bounds for a 95% confidence interval (H0: r = 0), the upper Ur and lower Lr bounds determined by bootstrap resampling, the probability P of a one-tailed test for positive autocorrelation, and the x-intercept are shown across all distance classes.
(DOCX)

S6 TableMeta-analysis data including each study used, the FST slope calculated as $\beta \;=\;\frac{\Delta F_{ST}}{\Delta Distance}$ and the spatial pattern identified in each study.(DOCX)

S7 Table Distance predictions according to *F*_ST_ based on meta-analysis data. (DOCX)
