# Supplementary figures and images for: Understanding the Spatial Scale of Genetic Connectivity at Sea: Unique Insights from a Land Fish and a Meta-Analysis
Source: PLoS One. 2016 May 19;11(5):e0150991. doi: 10.1371/journal.pone.0150991 (PMC4873183; doi:10.1371/journal.pone.0150991)

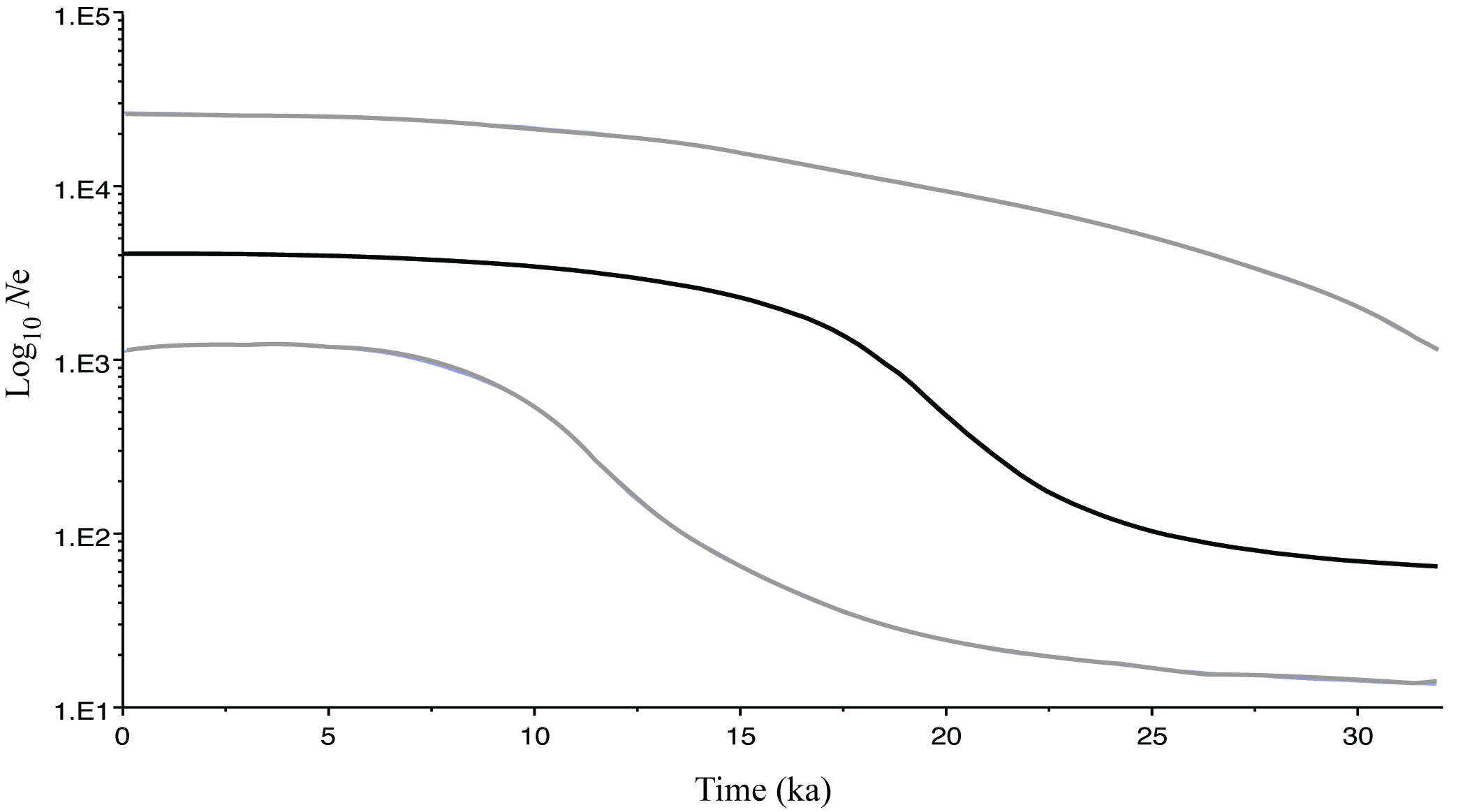

Supplement: S2 Fig — The thick black line is the median estimate of the log10 of the effective population size, and the thin grey lines are the 95% higher posterior density. (TIF) [file pone.0150991.s002.tif]

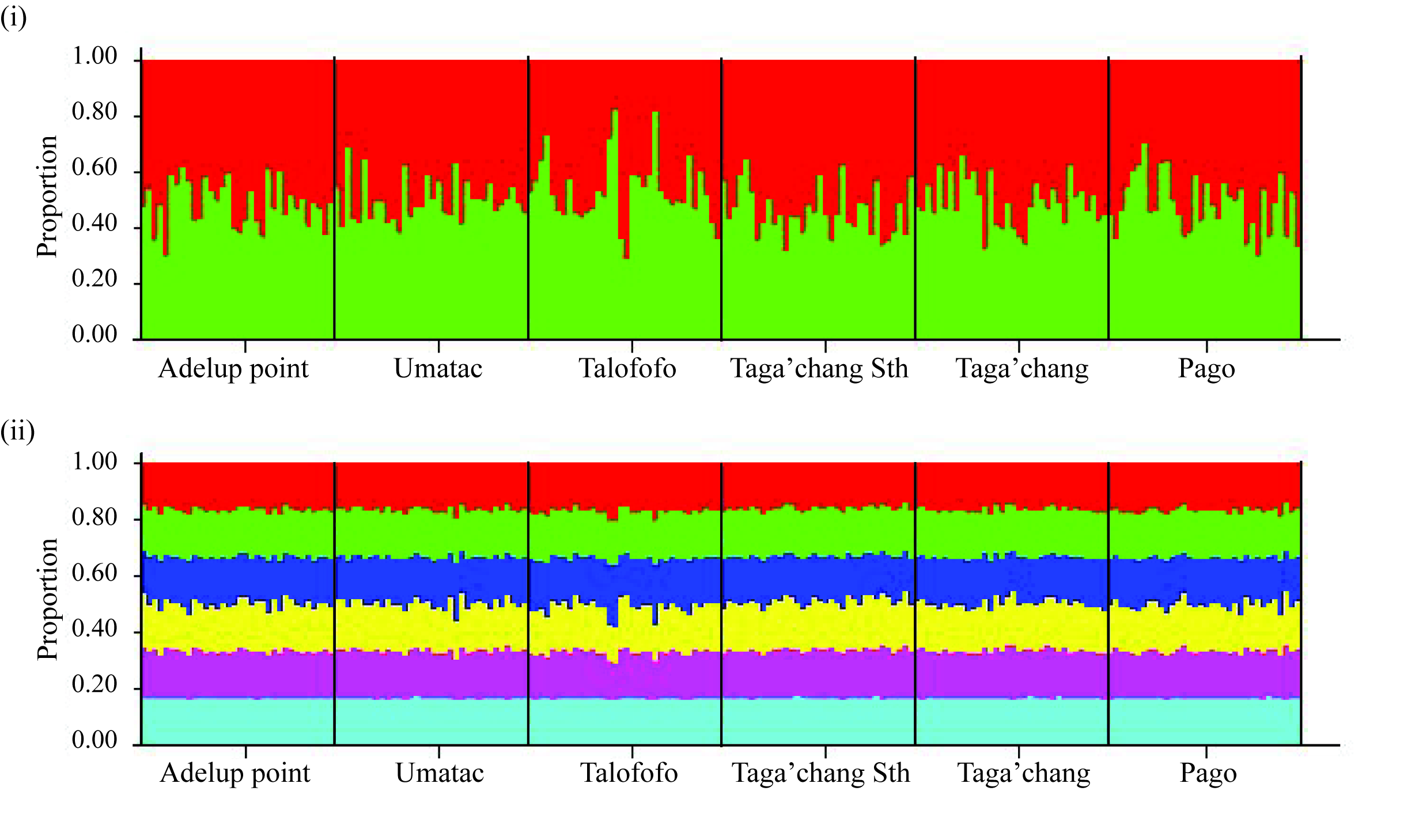

Supplement: S3 Fig — Individuals are grouped by sampling location and each individual is represented by one vertical line broken into K coloured segments, with the lengths being proportional to the K inferred cluster. (TIF) [file pone.0150991.s003.tif]
