# Supplementary material for: Understanding the Spatial Scale of Genetic Connectivity at Sea: Unique Insights from a Land Fish and a Meta-Analysis
Source: PLoS One. 2016 May 19;11(5):e0150991. doi: 10.1371/journal.pone.0150991 (PMC4873183; doi:10.1371/journal.pone.0150991)
Supplement: S1 Table — Types of fluorescence used to label forward primers are indicated with the primer sequence (FAM, NED, PET, VIC). NA, number of alleles. (DOCX) [file pone.0150991.s004.docx]

**S1 Table.** **Characterisation of the 17 polymorphic microsatellite loci for *Alticus arnoldorum* (N=204) and multiplex panel design.** Types of fluorescence used to label forward primers are indicated with the primer sequence (FAM, NED, PET, VIC). N_A_, number of alleles.

| **Locus** | **Primer sequence 5’-3’** | **Motif** | **Size (bp)** | **N_A_** |
| --- | --- | --- | --- | --- |
|  | **Panel 1** |  |  |  |
| **AAR01** | F: GTGACAGACAGGGGTTTGCT^PET^  R: TTGGTCATCAAGGACTGCAA | TG | 141-191 | 20 |
| **AAR02** | F: GCTCCCAATGGATACACTGAA^FAM^  R: TCCGCTGCTTTACCAATACC | TG | 136-148 | 4 |
| **AAR07** | F: GACATGTGAAGGCAGTCGTG^VIC^  R: TGAGAAGGACAAGCGGGTTA | CATT | 122-186 | 15 |
| **AAR10** | F: TCTTACTTGCTGAGCGTGGA^PET^  R: GAGTGGCTTTCAACATCCGT | AC | 331-355 | 11 |
| **AAR13** | F: TGAATGTTGTCTGACGGAACA^FAM^  R: CGGGAGGCGTGTACTGTATC | TC | 254-280 | 6 |
| **AAR16** | F: GCAGGGCTGCAATGATAACT^NED^  R: CTGCACACGAACACTGAAGC | GATA | 134-218 | 21 |
|  | **Panel 2** |  |  |  |
| **AAR04** | F: CAGCAGAGCCTGTCAACAAA^VIC^  R: GGTGGATCGTGACATTAGCC | ATAA | 258-338 | 21 |
| **AAR08** | F: CCTGTTCTCCACCGTGAAAT^NED^  R: TGAAGTTAAGAATCAAAGATGAGGTG | CTAT | 126-240 | 31 |
| **AAR09** | F: TGTTATTGGAATGTACAGTATGTTTGC^VIC^  R: CTCATCGAAACAGCTGGATG | AGAT | 103-151 | 13 |
| **AAR11** | F: CGAGTAAGGTGCAGGAGGAG^NED^  R: GGAAGAACAAAGCTTCCTTGA | GA | 332-374 | 22 |
| **AAR15** | F: CGCTGACCTCCAAATTTCA^PET^  R: AACTGCAGTGCAACCTGCTA | TG | 148-192 | 16 |
| **AAR17** | F: TGGTCTACTGAACAGGGCTTC^FAM^  GATGGATGGTATGAACATGTCG | TCAC | 149-213 | 17 |
|  | Panel 3 |  |  |  |
| **AAR03** | F: TTATCGCTGACCATGCATTC^NED^  R: CACGTTCAGGACAATAACTCCA | TCAT | 112-150 | 8 |
| **AAR05** | F: AATGAGACCTGTCCTGGGTG^NED^  R: AGCCTGGTGCAGATTCAAAC | TGGA | 202-274 | 19 |
| **AAR06** | F: TGGTTTCTGTCTGAATTTCCC^VIC^  R: GACTGGAAATTTGACGTCCC | TATC | 158-246 | 20 |
| **AAR12** | F: GGTACCGATCATGGTGAAGG^PET^  R: TGGTGATAAAGGCTTCTGCC | CA | 243-267 | 12 |
| **AAR14** | F: CTGCCTGCAACTGTCATCTC^PET^  R: TTCCAAACAGGATGATGGTG | AC | 102-130 | 12 |
