# Supplementary material for: Understanding the Spatial Scale of Genetic Connectivity at Sea: Unique Insights from a Land Fish and a Meta-Analysis
Source: PLoS One. 2016 May 19;11(5):e0150991. doi: 10.1371/journal.pone.0150991 (PMC4873183; doi:10.1371/journal.pone.0150991)
Supplement: S2 Table — Na, number of alleles per locus; Ar, allelic richness; FIS, Wrights inbreeding coefficient; HW Obs, Hardy-Weinberg observed heterozygosity; HW Exp. Hardy-Weinberg expected heterozygosity; and HW p-value, Hardy-Weinberg P-value; *, significant after sequential Bonferonni correction. (DOCX) [file pone.0150991.s005.docx]

**S2 Table. Descriptive statistics and diversity indices for each population per locus**. *N*_a_, number of alleles per locus; A_r_, allelic richness; *F*_IS,_ Wrights inbreeding coefficient; HW Obs, Hardy-Weinberg observed heterozygosity; HW Exp. Hardy-Weinberg expected heterozygosity; and HW p-value, Hardy-Weinberg P-value; *, significant after sequential Bonferonni correction

|  | | **Locus** |  |  |  |  |  |  |  |  |  |  |  |  |  |  |  |  |
| --- | --- | --- | --- | --- | --- | --- | --- | --- | --- | --- | --- | --- | --- | --- | --- | --- | --- | --- |
| **Population** | | **AR01** | **AR02** | **AR03** | **AR04** | **AR05** | **AR06** | **AR07** | **AR08** | **AR09** | **AR10** | **AR11** | **AR12** | **AR13** | **AR14** | **AR15** | **AR16** | **AR17** |
| **Adelup Point** | ***N*_a_** | 9 | 4 | 6 | 18 | 14 | 13 | 10 | 20 | 11 | 7 | 11 | 8 | 3 | 9 | 8 | 15 | 14 |
|  | **A_r_** | 8.554 | 3.686 | 5.833 | 17.329 | 13.518 | 12.772 | 9.664 | 18.991 | 10.83 | 6.833 | 10.896 | 7.828 | 3 | 8.558 | 7.813 | 14.894 | 13.37 |
|  | ***F*_IS_** | 0.145 | 0.49 | -0.076 | 0.14 | -0.006 | 0.547 | 0.174 | -0.066 | 0.032 | 0.078 | 0.033 | -0.142 | -0.031 | -0.091 | 0.304 | 0.079 | 0.039 |
|  | **HW Obs.** | 0.676 | 0.059 | 0.676 | 0.794 | 0.912 | 0.412 | 0.529 | 0.971 | 0.853 | 0.618 | 0.794 | 0.794 | 0.382 | 0.794 | 0.559 | 0.853 | 0.853 |
|  | **HE Exp.** | 0.790 | 0.115 | 0.6230 | 0.922 | 0.906 | 0.901 | 0.640 | 0.912 | 0.881 | 0.669 | 0.821 | 0.697 | 0.371 | 0.729 | 0.800 | 0.925 | 0.887 |
|  | **HW P-value** | 0.109 | 0.030 | 0.689 | 0.004 | 0.348 | *0.000 | 0.013 | 0.998 | 0.173 | 0.135 | 0.267 | 0.936 | 0.394 | 0.782 | *0.002 | 0.140 | 0.484 |
| **Umatic** | ***N*_a_** | 7 | 3 | 6 | 16 | 15 | 14 | 9 | 19 | 10 | 7 | 15 | 8 | 3 | 8 | 10 | 16 | 12 |
|  | **A_r_** | 6.705 | 2.960 | 5.813 | 15.389 | 14.369 | 13.117 | 8.958 | 18.141 | 9.851 | 6.833 | 14.076 | 7.791 | 2.853 | 7.853 | 9.622 | 15.222 | 11.683 |
|  | ***F*_IS_** | 0.069 | -0.031 | -0.178 | 0.049 | 0.074 | 0.472 | 0.040 | 0.041 | 0.018 | 0.108 | 0.082 | 0.053 | 0.002 | 0.065 | 0.203 | 0.036 | -0.023 |
|  | **HW Obs.** | 0.618 | 0.118 | 0.765 | 0.882 | 0.824 | 0.471 | 0.706 | 0.882 | 0.848 | 0.647 | 0.794 | 0.647 | 0.441 | 0.765 | 0.636 | 0.882 | 0.912 |
|  | **HE Exp.** | 0.663 | 0.114 | 0.651 | 0.927 | 0.889 | 0.885 | 0.735 | 0.919 | 0.863 | 0.724 | 0.864 | 0.683 | 0.442 | 0.817 | 0.796 | 0.914 | 0.892 |
|  | **HW P-value** | 0.044 | 1.000 | 0.409 | 0.152 | 0.348 | *0.000 | 0.089 | 0.621 | 0.821 | 0.840 | 0.504 | 0.413 | 1.000 | 0.683 | 0.156 | 0.680 | 0.959 |
| **Talofofo** | ***N*_a_** | 15 | 2 | 5 | 13 | 13 | 14 | 12 | 21 | 10 | 10 | 15 | 11 | 6 | 10 | 10 | 18 | 13 |
|  | **A_r_** | 13.801 | 1.987 | 4.879 | 12.552 | 12.622 | 13.415 | 11.367 | 20.698 | 10.000 | 9.519 | 14.930 | 10.827 | 5.703 | 9.830 | 9.730 | 17.075 | 12.839 |
|  | ***F*_IS_** | -0.087 | 1.000 | 0.082 | 0.211 | 0.091 | 0.529 | 0.171 | 0.077 | -0.053 | 0.294 | -0.005 | 0.185 | 0.076 | 0.018 | 0.267 | 0.020 | 0.070 |
|  | **HW Obs.** | 0.853 | 0.000 | 0.606 | 0.706 | 0.818 | 0.406 | 0.559 | 0.867 | 0.931 | 0.559 | 0.867 | 0.676 | 0.441 | 0.824 | 0.576 | 0.912 | 0.848 |
|  | **HE Exp.** | 0.786 | 0.060 | 0.660 | 0.892 | 0.899 | 0.855 | 0.673 | 0.937 | 0.885 | 0.788 | 0.862 | 0.828 | 0.477 | 0.838 | 0.782 | 0.930 | 0.911 |
|  | **HW P-value** | 0.140 | 0.016 | 0.421 | 0.100 | 0.472 | *0.000 | 0.039 | 0.327 | 0.872 | *0.000 | 0.216 | 0.068 | 0.016 | 0.169 | 0.005 | 0.182 | 0.293 |
| **Taga’chang Sth** | ***N*_a_** | 9 | 3 | 4 | 15 | 12 | 11 | 9 | 19 | 10 | 9 | 15 | 7 | 3 | 9 | 8 | 13 | 15 |
|  | **A_r_** | 8.502 | 2.851 | 4.000 | 14.497 | 11.683 | 10.864 | 8.558 | 17.759 | 9.899 | 8.686 | 14.352 | 6.833 | 2.980 | 8.683 | 7.684 | 12.661 | 14.658 |
|  | ***F*_IS_** | 0.294 | -0.035 | 0.102 | 0.330 | 0.037 | 0.515 | 0.147 | -0.042 | -0.019 | 0.316 | 0.026 | -0.010 | 0.056 | 0.034 | 0.272 | -0.035 | -0.028 |
|  | **HW Obs.** | 0.545 | 0.118 | 0.529 | 0.618 | 0.853 | 0.424 | 0.529 | 0.912 | 0.875 | 0.500 | 0.882 | 0.735 | 0.324 | 0.706 | 0.559 | 0.912 | 0.941 |
|  | **HE Exp.** | 0.769 | 0.114 | 0.589 | 0.918 | 0.885 | 0.868 | 0.619 | 0.875 | 0.859 | 0.728 | 0.905 | 0.728 | 0.342 | 0.730 | 0.765 | 0.881 | 0.916 |
|  | **HW P-value** | *0.002 | 1.000 | 0.365 | *0.000 | 0.362 | *0.000 | 0.004 | 0.591 | 0.681 | *0.001 | 0.407 | 0.258 | 0.376 | 0.392 | *0.001 | 0.658 | 0.220 |
| **Taga’chang** | ***N*_a_** | 9 | 3 | 7 | 18 | 12 | 13 | 10 | 20 | 11 | 6 | 13 | 9 | 3 | 8 | 7 | 16 | 14 |
|  | **A_r_** | 8.539 | 2.851 | 6.794 | 17.202 | 11.663 | 12.663 | 9.517 | 18.722 | 10.864 | 5.960 | 12.245 | 8.809 | 2.980 | 7.980 | 6.853 | 15.617 | 13.789 |
|  | ***F*_IS_** | 0.054 | 0.486 | -0.025 | 0.148 | 0.004 | 0.436 | 0.214 | 0.034 | -0.025 | 0.165 | -0.049 | -0.034 | -0.153 | 0.042 | 0.135 | -0.024 | 0.140 |
|  | **HW Obs.** | 0.735 | 0.059 | 0.647 | 0.794 | 0.882 | 0.500 | 0.500 | 0.882 | 0.909 | 0.529 | 0.912 | 0.824 | 0.471 | 0.794 | 0.647 | 0.941 | 0.794 |
|  | **HE Exp.** | 0.777 | 0.114 | 0.632 | 0.930 | 0.885 | 0.881 | 0.634 | 0.913 | 0.888 | 0.633 | 0.870 | 0.797 | 0.409 | 0.829 | 0.746 | 0.919 | 0.921 |
|  | **HW P-value** | 0.832 | 0.090 | 0.113 | *0.000 | 0.496 | *0.000 | 0.015 | 0.429 | 0.223 | 0.392 | 0.522 | 0.598 | 0.798 | 0.299 | 0.039 | 0.832 | 0.026 |
| **Pago** | ***N*_a_** | 10 | 1 | 6 | 17 | 15 | 14 | 9 | 17 | 10 | 6 | 17 | 8 | 3 | 10 | 8 | 17 | 15 |
|  | **A_r_** | 9.392 | 1.000 | 5.833 | 16.201 | 14.115 | 13.894 | 8.936 | 16.088 | 9.890 | 5.975 | 15.759 | 7.843 | 3.000 | 9.813 | 7.559 | 16.345 | 14.532 |
|  | ***F*_IS_** | 0.244 | NA | -0.114 | 0.139 | -0.055 | 0.416 | 0.296 | 0.131 | 0.005 | 0.038 | 0.071 | -0.080 | -0.013 | -0.002 | 0.377 | 0.061 | 0.138 |
|  | **HW Obs.** | 0.588 | mono | 0.765 | 0.794 | 0.941 | 0.529 | 0.471 | 0.794 | 0.844 | 0.529 | 0.765 | 0.765 | 0.500 | 0.853 | 0.471 | 0.853 | 0.794 |
|  | **HE Exp.** | 0.775 | mono | 0.687 | 0.921 | 0.892 | 0.901 | 0.666 | 0.912 | 0.848 | 0.550 | 0.822 | 0.709 | 0.494 | 0.852 | 0.752 | 0.907 | 0.920 |
|  | **HW P-value** | 0.024 | NA | 0.828 | 0.008 | 0.932 | *0.000 | 0.004 | 0.136 | 0.943 | 0.611 | 0.664 | 0.349 | 0.889 | 0.172 | *0.000 | 0.318 | 0.122 |
