# Supplementary material for: Understanding the Spatial Scale of Genetic Connectivity at Sea: Unique Insights from a Land Fish and a Meta-Analysis
Source: PLoS One. 2016 May 19;11(5):e0150991. doi: 10.1371/journal.pone.0150991 (PMC4873183; doi:10.1371/journal.pone.0150991)
Supplement: S3 Table — No comparisons were significantly different. (DOCX) [file pone.0150991.s006.docx]

**S3 Table. Pairwise Φ_ST_ comparisons for the 7 sampled populations of *Alticus arnoldorum*.** No comparisons were significantly different.

|  | **Adelup Point** | **Umatic** | **Talofofo** | **Taga’chang Sth** | **Taga’chang** | **Pago** |
| --- | --- | --- | --- | --- | --- | --- |
| **Adelup Point** | 0 |  |  |  |  |  |
| **Umatic** | -0.01754 | 0 |  |  |  |  |
| **Talofofo** | -0.03434 | -0.01183 | 0 |  |  |  |
| **Taga’chang Sth** | 0.00608 | 0.00967 | -0.00924 | 0 |  |  |
| **Taga’chang** | -0.01439 | -0.00229 | -0.02461 | -0.01231 | 0 |  |
| **Pago** | 0.01366 | 0.01727 | -0.00181 | -0.01154 | -0.02362 | 0 |
