# Supplementary material for: Understanding the Spatial Scale of Genetic Connectivity at Sea: Unique Insights from a Land Fish and a Meta-Analysis
Source: PLoS One. 2016 May 19;11(5):e0150991. doi: 10.1371/journal.pone.0150991 (PMC4873183; doi:10.1371/journal.pone.0150991)
Supplement: S4 Table — (DOCX) [file pone.0150991.s007.docx]

**S4 Table. Pairwise F_ST_ comparisons for the 7 sampled populations of *Alticus arnoldorum*, (i) total data set, (ii) data set excluding null alleles (*P≤0.05 after bonferroni correction).**

|  | | **Adelup Point** | **Umatic** | **Talofofo** | **Taga’chang Sth** | **Taga’chang** | **Pago** |
| --- | --- | --- | --- | --- | --- | --- | --- |
| (i) | **Adelup Point** | 0 |  |  |  |  |  |
|  | **Umatic** | 0.00662* | 0 |  |  |  |  |
|  | **Talofofo** | 0.00671 | 0.00228 | 0 |  |  |  |
|  | **Taga’chang Sth** | 0.00382 | 0.0064 | 0.00736* | 0 |  |  |
|  | **Taga’chang** | 0.00701* | 0.00248 | 0.00189 | 0.0062 | 0 |  |
|  | **Pago** | 0.00686* | 0.00409 | 0.00503 | 0.00948* | 0.0008 | 0 |
| (ii) | **Adelup Point** | 0 |  |  |  |  |  |
|  | **Umatic** | 0.00327 | 0 |  |  |  |  |
|  | **Talofofo** | 0.00421 | 0.00383 | 0 |  |  |  |
|  | **Taga’chang Sth** | -0.00054 | 0.00641 | 0.00857* | 0 |  |  |
|  | **Taga’chang** | 0.00938* | -0.00084 | 0.00262 | 0.01339* | 0 |  |
|  | **Pago** | 0.01027* | -0.00061 | 0.00127 | 0.01534* | 0.00435 | 0 |
