# Supplementary material for: Understanding the Spatial Scale of Genetic Connectivity at Sea: Unique Insights from a Land Fish and a Meta-Analysis
Source: PLoS One. 2016 May 19;11(5):e0150991. doi: 10.1371/journal.pone.0150991 (PMC4873183; doi:10.1371/journal.pone.0150991)
Supplement: S5 Table — The number of pairwise comparisons, N, correlation, r, upper U and lower L bounds for a 95% confidence interval (H0: r = 0), the upper Ur and lower Lr bounds determined by bootstrap resampling, the probability P of a one-tailed test for positive autocorrelation, and the x-intercept are shown across all distance classes. (DOCX) [file pone.0150991.s008.docx]

**S5 Table:** Spatial Autocorrelation analysis for the microsatellite data set excluding putative null alleles. The number of pairwise comparisons, *N*, correlation, r, upper U and lower L bounds for a 95% confidence interval (H0: r = 0), the upper Ur and lower Lr bounds determined by bootstrap resampling, the probability P of a one-tailed test for positive autocorrelation, and the x-intercept are shown across all distance classes.

| **Distance class** | **0** | **5** | **10** | **15** | **20** | **25** | **30** | **35** | **40** | **45** | **50** | **55** | **60** | **65** | **70** | **75** | **80** | **85** | **90** | **95** | **100** |
| --- | --- | --- | --- | --- | --- | --- | --- | --- | --- | --- | --- | --- | --- | --- | --- | --- | --- | --- | --- | --- | --- |
| ***N*** | 3366 | 3468 | 2312 | 1190 | 34 | 34 | 0 | 1122 | 0 | 2244 | 2278 | 0 | 0 | 0 | 0 | 0 | 1156 | 0 | 2346 | 1156 | 0 |
| **r** | 0.009 | -0.006 | -0.002 | 0.002 | 0.042 | -0.001 | 0.005 | -0.006 | 0.005 | 0.000 | 0.001 | 0.005 | 0.005 | 0.005 | 0.005 | 0.005 | -0.004 | 0.005 | 0.004 | -0.010 | 0.005 |
| **U** | 0.004 | 0.003 | 0.004 | 0.009 | 0.043 | 0.043 | 0.005 | 0.009 | 0.005 | 0.004 | 0.005 | 0.005 | 0.005 | 0.005 | 0.005 | 0.005 | 0.007 | 0.005 | 0.004 | 0.006 | 0.005 |
| **L** | -0.005 | -0.005 | -0.005 | -0.008 | -0.049 | -0.040 | 0.005 | -0.010 | 0.005 | -0.006 | -0.006 | 0.005 | 0.005 | 0.005 | 0.005 | 0.005 | -0.010 | 0.005 | -0.005 | -0.008 | 0.005 |
| **P(r-rand >= r-data)** | 0.010 | 1.000 | 0.810 | 0.290 | 0.040 | 0.530 | 1.000 | 0.930 | 1.000 | 0.410 | 0.360 | 1.000 | 1.000 | 1.000 | 1.000 | 1.000 | 0.830 | 1.000 | 0.090 | 1.000 | 1.000 |
| **Ur** | 0.015 | -0.001 | 0.004 | 0.009 | 0.083 | 0.037 | 0.000 | 0.002 | 0.000 | 0.006 | 0.007 | 0.000 | 0.000 | 0.000 | 0.000 | 0.000 | 0.004 | 0.000 | 0.009 | -0.003 | 0.000 |
| **Lr** | 0.004 | -0.012 | -0.007 | -0.005 | -0.017 | -0.043 | 0.000 | -0.014 | 0.000 | -0.007 | -0.006 | 0.000 | 0.000 | 0.000 | 0.000 | 0.000 | -0.011 | 0.000 | -0.002 | -0.019 | 0.000 |
| **Intercept** | 3.083 |  |  |  |  |  |  |  |  |  |  |  |  |  |  |  |  |  |  |  |  |
