# Supplementary material for: Understanding the Spatial Scale of Genetic Connectivity at Sea: Unique Insights from a Land Fish and a Meta-Analysis
Source: PLoS One. 2016 May 19;11(5):e0150991. doi: 10.1371/journal.pone.0150991 (PMC4873183; doi:10.1371/journal.pone.0150991)
Supplement: S6 Table — (DOCX) [file pone.0150991.s009.docx]

**S6 Table. Meta-analysis data including each study, *F*_ST_ slope calculated as *β* =**$\frac{\boldsymbol{\Delta}\boldsymbol{F}_{\boldsymbol{ST}}}{\boldsymbol{\Delta Distance}}$ **and the spatial pattern identified in each study.**

| **Species** | **Order** | **Spatial Pattern** | ***F*_ST_ Slope** | **IBD present/absent/not specified** | **Explanation of spatial pattern** | **Reference** |
| --- | --- | --- | --- | --- | --- | --- |
| *Merlangius merlangus* | Gadiformes | Low level of genetic structuring | -4.20 × 10^-6^ | No IBD | Oceanographic patterns | Charrier et al. (2007) *Mar. Ecol. Prog. Ser.* 330: 201-211 |
| *Gadus morhua* | Gadiformes | Low level of genetic structuring | 0.0003 | No IBD | Local topography | Jorde et al. (2007) *Mar. Ecol. Prog. Ser.* 343:229-237 |
| *Sardina pilchardus* | Clupeiformes | Low level of genetic structuring | 2.47 × 10^-7^ | IBD | Some contemporary gene flow | Gonzalez & Zardoya (2007) *BMC Evol. Biol.* 7:197 |
| *Thalassoma bifasciatum* | Perciformes | No spatial population structure | 2.41 × 10^-6^ | No specified | Historical demographic processes | Haney et al. (2007) *Heredity* 98: 294-302 |
| *Psetta maxima* | Pleuronectiformes | No spatial population structure | 8.69 × 10^-6^ | No IBD | Migration | Florin and Hoglund (2007) *Mol. Ecol.* 16:115-126 |
| *Clupea harengus* | Clupeiformes | Spatial population structure | 1.06 × 10^-6^ | IBD | IBD | Larsson et al. (2007) *Mol. Ecol.* 16:1135-1147 |
| *Pagrus pagrus* | Perciformes | Spatial population structure | 2.09 × 10^-5^ | Not specified | Large scale differentiation, regional homogeneity | Ball et al. (2007) *Mar. Biol.* 150:1321-1332 |
| *Oncorhynchus nerka* | Salmoniformes | Spatial population structure | -0.006 | Not specified | Natal homing, selection against ‘strays’ | Lin et al. (2008) *Heredity*. 101:341-350 |
| *Salmo salar L.* | Salmoniformes | Spatial population structure | 0.0003 | IBD | Geographic separation | Dillane et al. (2008) *Mol. Ecol.* 17:4786-4800 |
| *Raja clavata* | Rajiformes | Low level of genetic structuring | 0 | Not specified | Gene flow | Chevolot et al. (2008) *Heredity* 101:120-126 |
| *Thunnus obesus* | Perciformes | No spatial population structure | 1.09 × 10^-6^ | No IBD | Panmixia | Gonzalez et al. (2008) *BMC Evol. Biol.* 8:252 |
| *Fundulus heteroclitus* | Cyprinodontiformes | Spatial population structure | 8.72 × 10^-5^ | IBD | IBD | Duvernell et al. (2008) *Mol. Ecol.* 17:1344-1360 |
| *Platichthys flesus* | Pleuronectiformes | Spatial population structure | 1.96 × 10^-5^ | IBD | IBD | Florin & Hoglund (2008) *Heredity* 101:27-38 |
| *Acanthochromis polyacanthus* | Perciformes | Spatial population structure | 0.0003 | IBD | IBD and historical colonization patterns | Miller-Sims et al. (2008) *Mol. Ecol.* 17:5036-5048 |
| *Negaprion acutidens* | Carcharhiniformes | Spatial population structure | -4.86 × 10^-6^ | No IBD | Biogeographic history | Schultz et al. (2008) *Mol. Ecol.* 17:5336-5348 |
| *Micromesistius poutassou* | Gadiformes | Spatial population structure | -0.0001 | No IBD | Hydrographic patterns, fish behaviour | Was et al. (2008) *ICES J. Mar. Sci.* 65:216-225 |
| *Mallotus villosus* | Osmeriformes | Spatial population structure | 9.36 × 10^-6^ | Not specified | Regional isolation | Praebel et al. (2008) *Mar. Ecol. Prog. Ser.* 360:189-199 |
| *Gadus morhua* | Gadiformes | Spatial population structure | 6.34 × 10^-6^ | Not specified | Biogeographic history | Pampoulie et al. (2008) *ICES J. Mar. Sci.* 65:65-71 |
| *Laterolabrax japonicus* | Perciformes | Spatial population structure | 2.74 × 10^-5^ | Not specified | Historical bottleneck | Jiang et al. (2008) *J. Appl. Ichthyol.* 24:180-186 |
| *Parma microlepis* | Perciformes | No spatial population structure | -2.57 × 10^-5^ | No IBD | High gene flow | Curley & Gillings (2009) *Mar. Biol.* 156:381-393 |
| *Gobionotothen gibberifrons* | Perciformes | No spatial population structure | 0 | Not specified | Gene flow | Matschiner et al (2009) *Mol. Ecol.* 18:2574-2587 |
| *Hoplostethus atlanticus* | Beryciformes | No spatial population structure | 1.93 × 10^-6^ | Not specified | Panmixia, dispersal | White et al. (2009) *Mol. Ecol.* 18:2563-2573 |
| *Gadus macrocephalus* | Gadiformes | Spatial population structure | 3.46 × 10^-6^ | IBD | Limited dispersal | Cunningham et al. (2009). *Can. J. Fish. Aquat. Sci.* 66:153-166 |
| *Stegastes partitus* | Perciformes | Spatial population structure | 1.09 × 10^-5^ | IBD | IBD and oceanographic and landscape features | Purcell et al. (2009) *J. Fish Biol.* 74:403-417 |
| *Oncorhynchus mykiss* | Salmoniformes | Spatial population structure | -0.005 | Not specified | Selection among populations | Pearse et al. (2009) *J. Heredity*. 100:515-525 |
| *Sebastes mentella* | Scorpaeniforme | Spatial population structure | 6.35 × 10^-7^ | No IBD | Depth segregation | Stefansson et al. (2009) *ICES J. Mar. Sci.* 66:680-690 |
| *Etheostoma caeruleum* | Perciformes | Spatial population structure | 6.33 × 10^-5^ | No IBD | Biogeographic history | Haponski et al. (2009) *J. Fish Biol.* 75:2244-2268 |
| *Sprattus sprattus* L. | Clupeiformes | Spatial population structure | 4.73 × 10^-5^ | No IBD | Habitat boundaries | Limborg et al. (2009) *Mar. Ecol. Prog. Ser.* 379:213-224 |
| *Mullus barbatus* | Perciformes | Spatial population structure | -0.005 | Not specified | Biogeography and habitat boundaries | Maggio et al. (2009) *ICES J. Mar. Sci.* 66:1883-1891 |
| *Lates calcarifer* | Perciformes | Spatial population structure | 0.0001 | Not specified | Geographic separation | Yue et al. (2009) *Aquaculture* 293:22-28 |
| *Stegastes partitus* | Perciformes | Low level of genetic structuring | -2.25 × 10^-6^ | No IBD | Habitat boundaries | Salas et al. (2010) *Mar. Biol.* 157:437-445 |
| *Stegastes partitus* | Perciformes | No spatial population structure | 3.91 × 10^-5^ | No IBD | “Sweepstake chance effect”, oceanography | Villegas-Sanchez et al. (2010) *Coral Reefs* 29:1023-1033 |
| *Chrysoblephus laticeps* | Perciformes | No spatial population structure | 1.70 × 10^-6^ | No IBD | High dispersal potential | Teske et al. (2010) *Mar. Biol.* 157-2029-2042 |
| *Coryphaena hippurus* | Perciformes | No spatial population structure | 1.73 × 10^-5^ | No IBD | Panmixia | Tripp-Valdez (2010) *Fish. Res.* 105:172-177 |
| *Syngnathus floridae* | Syngnathiformes | Spatial population structure | 1.79 × 10^-5^ | IBD | Low migration | Mobley et al. (2010) *J. Biogeog.* 37:1363-1377 |
| *Anarhichas lupus* | Perciformes | Spatial population structure | -7.84 × 10^-7^ | IBD | Limited dispersal, biogeography | McCusker & Bentzen (2010) *Mol. Ecol.* 19:4228-4241 |
| *Syngnathus typhle* | Syngnathiformes | Spatial population structure |  | IBD | Biogeographic history | Wilson & Veraguth (2010) *Mol. Ecol.* 19:4535-4553 |
| *Coryphaenoides rupestris* | Gadiformes | Spatial population structure | 0 | No IBD | Biographic history | White et al. (2010) *Mol. Ecol.* 19:216-226 |
| *Pleuronectes platessa* | Pleuronectiformes | Spatial population structure | 1.51 × 10^-5^ | No IBD | Bathymetric and hydrographic barriers | Was et al. (2010) *Mar. Biol.* 157:447-462 |
| *Thunnus thynnus* | Perciformes | Spatial population structure | 0 | Not specified | Demographic changes | Riccioni et al. (2010) *PNAS*. 107:2102-2107 |
| *Urobatis halleri* | Rajiformes | Spatial population structure | -7.58 × 10^-6^ | Not specified | Founder event | Plank et al. (2010) *J. Fish Biol.* 77:329-340 |
| *Gadus macrocephalus* | Gadiformes | Spatial population structure | -8.62 × 10^-5^ | Not specified | Biogeographic history | Kim et al. (2010) *Fish Sci.* 76:595-603 |
| *Scomberomorus semifasciatus* | Perciformes | Low levels of genetic structuring | 4.24 × 10^-5^ | No IBD | Habitat boundaries | Broderick et al. (2011) *J. Fish Biol.* 79:633-661 |
| *Merluccius hubbsi* | Gadiformes | Low levels of genetic structuring | 1.51 × 10^-6^ | Not specified | Habitat boundaries | Machado-Schiaffino et al. (2011) *Biol. Conserv.* 144:330-338 |
| *Merluccius bilinearis* | Gadiformes | Low levels of genetic structuring | 1.29 × 10^-6^ | Not specified | Habitat boundaries | Machado-Schiaffino et al. (2011) *Biol. Conserv.* 144:330-338 |
| *Coryphaenoides brevibarbis* | Gadiformes | No spatial population structure | 5.67 × 10^-6^ | Not specified | Habitat boundaries | White et al. (2011) *Mar. Ecol. Prog. Ser.* 434:155-164 |
| *Antimora rostrata* | Gadiformes | No spatial population structure | 7.16 × 10^-6^ | No IBD | Panmixia, large effective population sizes | White et al., (2011) *Heredity*. 106:690-699 |
| *Zebrasoma flavescens* | Perciformes | Spatial population structure | -2.04 × 10^-6^ | IBD | Reduced larval exchange with distance | Eble et al. (2011) *Mar. Ecol. Prog. Ser.* 428:245-258 |
| *Sphyrna lewini* | Carcharhiniformes | Spatial population structure | -1.20 × 10^-6^ | IBD | Population decline | Nance et al. (2011) *Plos One*. 6:e21459 |
| *Epinephelus marginatus* | Perciformes | Spatial population structure | 1.02 × 10^-5^ | No IBD | Oceanographic patterns | Schunter et al. (2011) *J. Exp. Mar. Biol. Ecol.* 401:126-133 |
| *Verasper variegatus* | Pleuronectiformes | Spatial population structure | 5.61 × 10^-6^ | Not specified | Habitat boundaries | Sekino et al. (2011) *Conserv. Genet*. 12:139-159 |
| *Sparus aurata* | Perciformes | Spatial population structure | 0.0006 | Not specified | Habitat boundaries | Segvic-Bubic et al. (2011) *Aquaculture.* 318:309-315 |
| *Sprattus sprattus* | Clupeiformes | Spatial population structure | 4.72 × 10^-6^ | Not specified | Geographic separation | Glover et al. (2011) *ICES J. Mar. Sci.* 68:2145-2151 |
| *Scarus rubroviolaceus* | Perciformes | Spatial population structure | 5.67 × 10^-6^ | Not specified | Biogeographic history | Fitzpatrick et al. (2011) *Mol. Ecol.* 20:219-234 |
| *Oncorhynchus nerka* | Salmoniformes | Low level of genetic structuring | -0.00006 | Not specified | Translocations among hatcheries | Yamamoto et al. (2011) *J. Fish. Biol.* 79:1340-1349 |
| *Macquaria colonorum* | Perciformes | No spatial population structure | -3.52 × 10^-5^ | No IBD | Oceanographic patterns | Shaddick et al. (2011) *Can. J. Fish. Aquat. Sci.* 68:304-318 |
| *Salmo salar* | Salmoniformes | Spatial population structure | 0.0001 | IBD | Geological substrate, river length | Perrier et al. (2011) *Mol. Ecol.* 20:4231-4245 |
| *Osmerus mordax* | Osmeriformes | Spatial population structure | 0.002 | Not specified | Biogeographic history | Bradbury et al. (2011) *Mar. Ecol. Prog. Ser.* 438:207-218 |
