# Supplementary material for: Understanding the Spatial Scale of Genetic Connectivity at Sea: Unique Insights from a Land Fish and a Meta-Analysis
Source: PLoS One. 2016 May 19;11(5):e0150991. doi: 10.1371/journal.pone.0150991 (PMC4873183; doi:10.1371/journal.pone.0150991)
Supplement: S7 Table — (DOCX) [file pone.0150991.s010.docx]

**S7 Table. Distance predictions according to *F*_ST_ based on meta-analysis data.**

| ***F*_ST_** | **Median distance (km)** | **Median CI lower (km)** | **Median CI upper (km)** |
| --- | --- | --- | --- |
| 0.01 | 701 | 211 | 1290 |
| 0.03 | 1264 | 292 | 2053 |
| 0.05 | 2140 | 306 | 4066 |
| 0.07 | 3015 | 633 | 5955 |
| 0.09 | 3151 | 879 | 6757 |
| 0.11 | 3848 | 728 | 8752 |
| 0.13 | 4545 | 763 | 9964 |
| 0.15 | 5242 | 810 | 11691 |
| 0.17 | 5938 | 793 | 13785 |
| 0.19 | 6635 | 776 | 15147 |
| 0.21 | 7332 | 958 | 19822 |
| 0.23 | 8029 | 1879 | 18817 |
| 0.25 | 10645 | 1870 | 24094 |
